# Characterization of novel Bovine Leukemia Virus (BLV) antisense transcripts by deep sequencing reveals constitutive expression in tumors and transcriptional interaction with viral microRNAs

**DOI:** 10.1186/s12977-016-0267-8

**Published:** 2016-05-03

**Authors:** Keith Durkin, Nicolas Rosewick, Maria Artesi, Vincent Hahaut, Philip Griebel, Natasa Arsic, Arsène Burny, Michel Georges, Anne Van den Broeke

**Affiliations:** Unit of Animal Genomics, GIGA-R, Université de Liège (ULg), 4000 Liège, Belgium; Laboratory of Experimental Hematology, Institut Jules Bordet, Université Libre de Bruxelles (ULB), 1000 Brussels, Belgium; Vaccine and Infectious Disease Organization, University of Saskatchewan, Saskatoon, S7N 2Z4 Canada; School of Public Health, University of Saskatchewan, Saskatoon, S7N 2Z4 Canada

**Keywords:** Bovine Leukemia Virus, Deltaretrovirus, Noncoding RNA, Leukemogenesis, High throughput sequencing, RNA-seq, Oxford Nanopore minION

## Abstract

**Background:**

Bovine Leukemia Virus (BLV) is a deltaretrovirus closely related to the Human T cell leukemia virus-1 (HTLV-1). Cattle are the natural host of BLV where it integrates into B-cells, producing a lifelong infection. Most infected animals remain asymptomatic but following a protracted latency period about 5 % develop an aggressive leukemia/lymphoma, mirroring the disease trajectory of HTLV-1. The mechanisms by which these viruses provoke cellular transformation remain opaque. In both viruses little or no transcription is observed from the 5′LTR in tumors, however the proviruses are not transcriptionally silent. In the case of BLV a cluster of RNA polymerase III transcribed microRNAs are highly expressed, while the HTLV-1 antisense transcript *HBZ* is consistently found in all tumors examined.

**Results:**

Here, using RNA-seq, we demonstrate that the BLV provirus also constitutively expresses antisense transcripts in all leukemic and asymptomatic samples examined. The first transcript (AS1) can be alternately polyadenylated, generating a transcript of ~600 bp (AS1-S) and a less abundant transcript of ~2200 bp (AS1-L). Alternative splicing creates a second transcript of ~400 bp (AS2). The coding potential of AS1-S/L is ambiguous, with a small open reading frame of 264 bp, however the transcripts are primarily retained in the nucleus, hinting at a lncRNA-like role. The AS1-L transcript overlaps the BLV microRNAs and using high throughput sequencing of RNA-ligase-mediated (RLM) 5′RACE, we show that the RNA-induced silencing complex (RISC) cleaves AS1-L. Furthermore, experiments using altered BLV proviruses with the microRNAs either deleted or inverted point to additional transcriptional interference between the two viral RNA species.

**Conclusions:**

The identification of novel viral antisense transcripts shows the BLV provirus to be far from silent in tumors. Furthermore, the consistent expression of these transcripts in both leukemic and nonmalignant clones points to a vital role in the life cycle of the virus and its tumorigenic potential. Additionally, the cleavage of the AS1-L transcript by the BLV encoded microRNAs and the transcriptional interference between the two viral RNA species suggest a shared role in the regulation of BLV.

**Electronic supplementary material:**

The online version of this article (doi:10.1186/s12977-016-0267-8) contains supplementary material, which is available to authorized users.

## Background

The deltaretrovirus Bovine Leukemia Virus (BLV), like its close relative Human T-cell leukemia virus (HTLV-1), produces a chronic infection in its natural host (cattle and water buffalo), with most infected individuals remaining asymptomatic and about 5 % going on to develop leukemia/lymphoma [1]. In humans the time between infection and the onset of Adult T cell leukemia/lymphoma (ATL) generally spans several decades [2], while in cattle several years separate infection from progression to B-cell leukemia/lymphoma [3]. BLV infections are common in cattle throughout the world (with the exception of most European countries, the result of eradication programs), imposing significant economic costs on the livestock industry [4]. While not a natural host, it is also possible to experimentally infect sheep with BLV and in contrast to the situation in cattle, all infected sheep generally develop B cell leukemia/lymphoma, about 20 months post infection [1].

BLV infects B-cells (in both cattle and sheep) and following an early and transient phase of horizontal replicative dissemination, primarily proliferates via polyclonal expansion, producing many long lived clones that can be tracked via their proviral integration site [5]. In a subset of infected individuals, for unknown reasons, one of these clones eventually begins to expand, producing an aggressive neoplasm that accumulates in the blood (B-cell leukemia) and/or organs (B-cell lymphoma). In both BLV and HTLV-1 the Tax protein has been seen as the principal actor in oncogenesis, especially as it is capable of driving cellular transformation [6]. However, the lack of Tax expression in the majority of ATLs [7] and BLV induced tumors [8], points to a more diverse cast of actors beyond Tax. In the case of HTLV-1 it has been evident for the last decade that the antisense product HTLV-1 basic leucine zipper factor (*HBZ*) plays a central role in the process of oncogenesis as it is found in 100 % of ATL cases examined [7, 9]. A large number of roles have been attributed to HBZ, with its inhibition of Tax mediated viral transcription postulated as a vital part of the viruses immune evasion strategy [7, 10]. Intriguingly, in addition to the protein encoded by *HBZ,* the *HBZ* mRNA has been implicated in supporting proliferation of ATL cells [11, 12]. In the case of BLV, no equivalent antisense transcript has been described, although it has recently been reported by us and others that the provirus encodes a cluster of non-canonical RNA polymerase III transcribed microRNAs on the positive strand. These viral microRNAs represent ~40 % of the microRNA pool in transformed B cells [13, 14]. Similar to HBZ in HTLV-1, the viral miRNAs are expressed in all BLV induced tumors examined to date, pointing to a vital role in the life cycle of the virus and cellular transformation [13].

The close relationship between BLV and HTLV-1 in genome organization and pathogenesis makes BLV an attractive model for investigating deltaretrovirus induced cancer. To further our understanding of the transcriptional landscape of BLV infected cells and to build on our previous work describing small noncoding RNAs we carried out high throughput RNA sequencing (RNA-seq) of total RNA from ovine and bovine BLV infected cells. Surprisingly, we observed a large number of reads mapping back to the BLV genome and found that these reads were the products of previously unidentified BLV antisense transcripts originating in the BLV 3′LTR. We present evidence that, like the case of HBZ, BLV antisense transcription appears to be a consistent feature of BLV infections. We also find that one of the transcripts is cleaved by the BLV microRNAs and discuss the possible role these transcripts play in the life cycle of BLV.

## Results

### Identification of two antisense transcripts encoded by BLV via deep sequencing

**Fig. 1:**
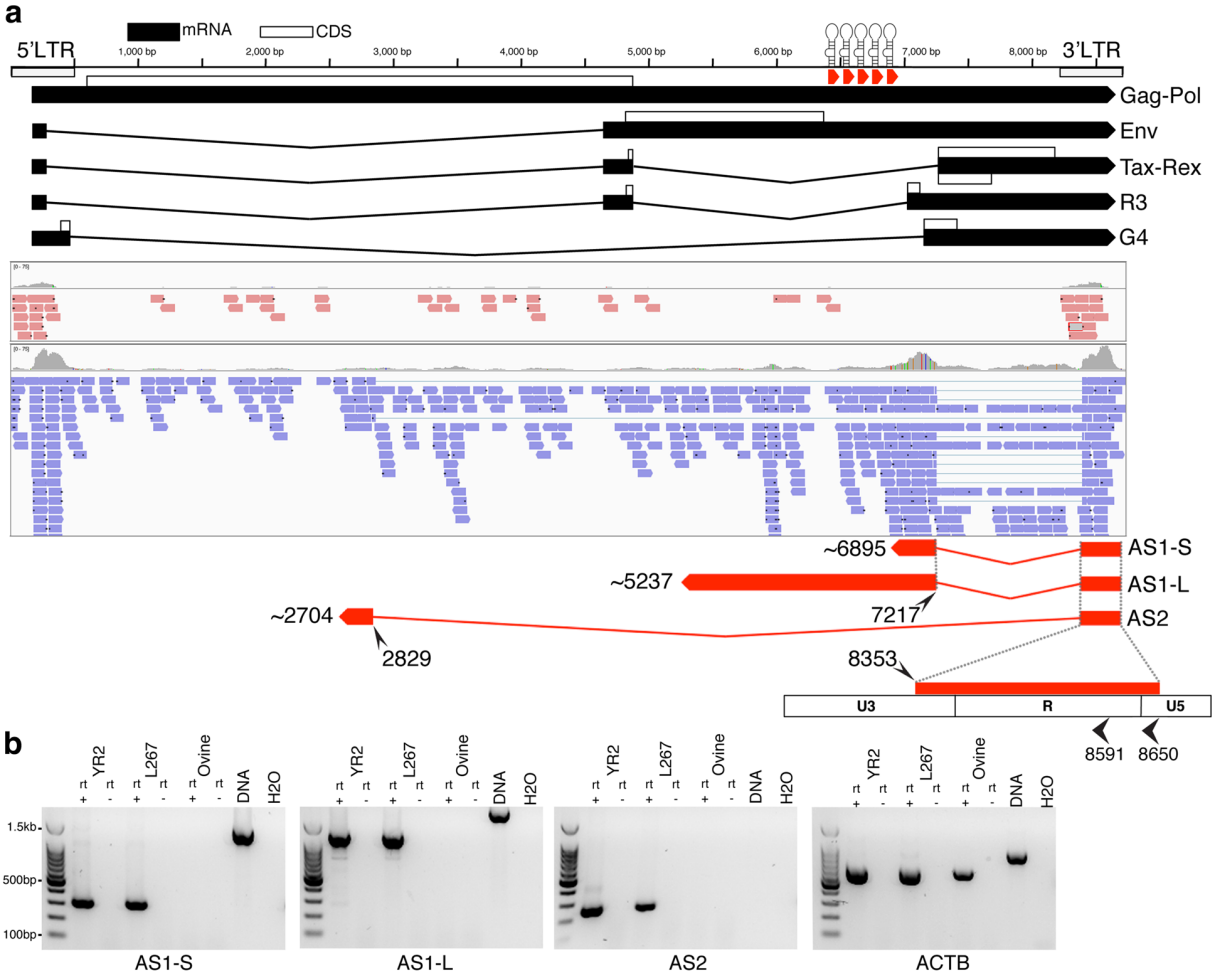
Identification of antisense transcription from the BLV provirus. **a** Ideogram showing location of the BLV sense transcripts originating in the 5′LTR as well as the BLV microRNAs. Below are shown the sense (*red*) and antisense (*blue*) reads mapping to the region from stranded RNA-seq of the L267 tumor B cell line. Based on observed antisense split reads and the results of 5′/3′RACE three BLV antisense transcripts were identified. All three share the same exon 1, with the 5′ end located primarily at either position 8591 or 8650. AS1-S ends as the transcript enters the BLV microRNA region and has a poly A tail. AS1-L extends beyond the BLV microRNA region and reads with a poly A tail can be observed at position ~5237. Finally, AS2 has a small exon 2 (~125 bp) and no poly A tail was observed in the majority of samples examined. **b** Rt-PCR using the YR2 and L267 cell lines and PBMCs from an uninfected sheep. Control DNA was derived from YR2. Primers used span the splice sites for AS1 and AS2. For AS1-L the reverse primer was upstream of the BLV microRNAs. Primers for Beta-actin (ACTB) were included as a positive control. (Rt+ reverse transcriptase positive, Rt− reverse transcriptase negative, base pair coordinates correspond to NCBI Accession: KT122858)

Previous work carried out by us and others had identified a cluster of ten microRNAs expressed from the BLV provirus genome utilizing a non-canonical microRNA biogenesis pathway involving RNA polymerase III [13, 14]. To better understand the pattern of RNA transcription during BLV infections we expanded our sequencing efforts beyond small RNAs and sequenced stranded RNA libraries produced from a variety of BLV infected samples. A total of 51 different samples were sequenced, including the BLV infected ovine B cell lines YR2 and L267, PBMCs from asymptomatic BLV infected sheep and BLV induced tumors isolated from both cattle and sheep. Viral transcripts and proteins originating from the 5′LTR are generally undetectable in tumors, the result of either genetic or epigenetic modifications of the provirus [8, 15, 16]. Stranded RNA-seq revealed a similar pattern with few sense reads observed and an absence of split reads corresponding to the spliced transcripts (Fig. 1a; Additional file 1: Table S1). The situation for the antisense coverage was significantly different, with a substantial number of antisense reads observed. Antisense read coverage was concentrated at the LTRs (as the LTRs are identical reads originating from the 3′LTR will also map to the 5′LTR and vice versa) and a ~300 bp region just downstream of the last BLV microRNA (miR-B5-3p). Additionally, the presence of split reads between base pairs 7217 and 8353 in the BLV genome pointed to a spliced transcript (denoted as Antisense 1, AS1). A second set of split reads were observed between base pairs 2829 and 8353, indicating the presence of a second transcript (denoted as Antisense 2, AS2). These putative splice sites do not overlap those found in previously described sense transcripts (Fig. 1a) and splice donor/acceptor sites matched the negative strand. PCR primers designed to span the putative splice sites of AS1 and AS2 showed amplification with cDNA derived from the YR2/L267 tumor cell lines and ovine/bovine tumors (Fig. 1b; Additional file 1: Fig. S1). PCR with a primer upstream of the BLV microRNAs and a primer in the first AS exon also showed amplification in YR2/L267 and the ovine/bovine tumors, demonstrating that the AS1 transcript could extend beyond the BLV microRNA region (AS1-L).

The number of viral antisense reads counted per million reads (CPM) from RNA sequencing of YR2/L267 and ovine/bovine tumors was broadly similar, ranging from 0.45 to 124.5 (mean = 15, median = 4.4). The CPM of viral sense reads ranged from 0 to 19.3 (mean = 1.7, median = 0.5) (Additional file 1: Table S1). The YR2_LTaxSN_ and L267_LTaxSN_ cell lines [8], which constitutively express 5′LTR dependent viral transcripts and proteins, in addition to the RNA pol III dependent microRNAs had antisense CPM of 29 and 4 respectively (Additional file 1: Table S1), suggesting that transcription from the 5′ and 3′LTRs of BLV, as well as from the RNA pol III promoters, are not mutually exclusive. When compared to other mRNAs the antisense transcripts have relatively modest expression levels, with ~73 and ~76 % of host genes having higher expression levels than AS1 and AS2 respectively. We also carried out RNA-seq on PBMCs isolated from 6 asymptomatic BLV infected sheep at 17 months post-inoculation. The proviral load in these animals ranged from 5.3 to 40.6 % (mean = 14.4 %; median = 9.3 %). High throughput sequencing (HTS) mapping of proviral integration sites showed that in five of these animals the largest clone (defined by percentage of reads observed at each insertion point) did not exceed 5 %. Antisense reads were detected in all 6 samples and at substantially higher numbers than sense reads. Additionally, the number of antisense reads mirrored the proviral load (Additional file 1: Table S1). We also observed consistent production of BLV microRNAs in these early stage samples (Additional file 1: Table S1). Collectively, these data support the conclusion that BLV antisense transcription, like BLV microRNA production, is a feature of both the asymptomatic and leukemic stages of BLV infection.

### Identification of 5′/3′ ends

To determine the 5′ and 3′ ends of both antisense transcripts rapid amplification of cDNA ends (RACE) was completed in combination with high throughput sequencing. For 5′RACE, total RNA from the cell line YR2 was used as template and mapping of the resultant reads showed that 42 % of AS1 and 46 % of AS2 transcripts started in U5 close to the R boundary at position 8650 (Fig. 1). A second site (8591) within the R region was also frequently utilized by both AS1 (27 %) and AS2 (21 %) (Fig. 1). Other less frequently used start sites are also listed (Additional file 1: Table S1).

**Fig. 2:**
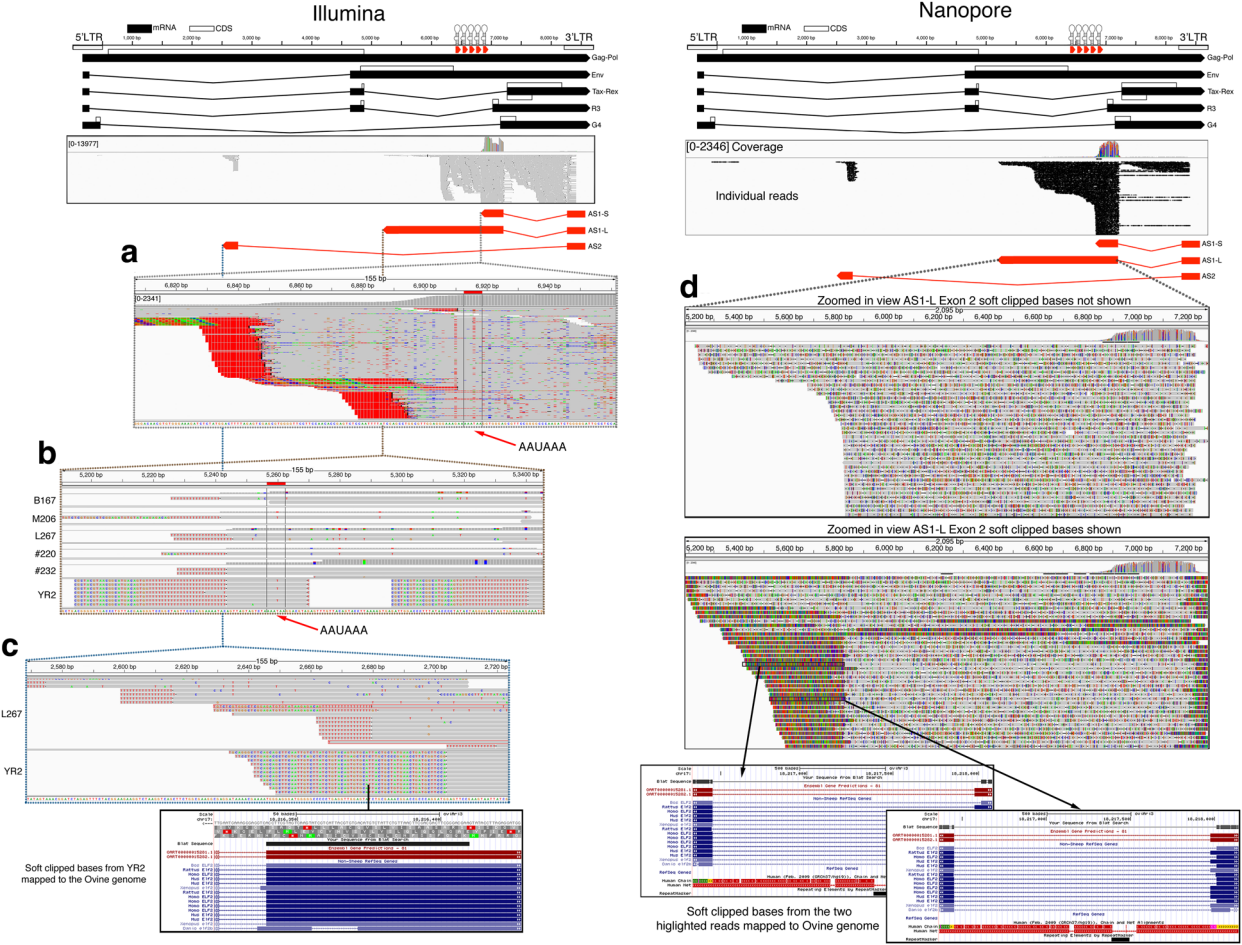
3′ ends of BLV antisense transcripts. Ideogram of the BLV provirus, with 3′RACE reads for L267 mapping to the BLV genome shown below. Potential 3′ ends of the BLV antisense transcripts are shown. **a** Zoomed in IGV screen shot showing a large cluster of reads with poly A tails (shown as T in IGV) adjacent to the end of miR-B5-3p. No signal position predominates, although the majority were located downstream (in the context of the AS transcript) of a canonical AAUAAA polyadenylation signal sequence (PAS). AS1 transcripts ending at this position were given the designation AS1-S. **b** In five of the libraries subjected to 3′RACE a small number of poly A reads were observed at position ~5237. This is located 20 bp downstream (in the context of the AS transcript) of a second canonical AAUAAA (PAS). Further, 3′RACE carried out on YR2 with the forward primer adjacent to this potential poly A site rather than the in AS1 exon 1 also showed polyadenylated products matching this position. **c** For AS2, only L267 showed any evidence of a poly A tail, however no canonical PAS was observed and no consistent poly A site was observed. In the remaining samples (also observed in many L267 reads) the AS2 transcript underwent splicing with the host genome. Shown are the soft clipped bases (the bases in the read that did not map to BLV) for YR2 mapped to the ovine genome with BLAT (http://genome.ucsc.edu/). These bases correspond to an exon of the ELF2 transcript ~5 kb upstream of the YR2 provirus integrated site. **d** Ideogram of the BLV provirus, with 3′RACE products from YR2 sequenced on a MinION instrument (Oxford Nanopore Technologies) and mapping to the BLV genome. Location of antisense transcripts are also shown. As with Illumina sequencing the majority of the reads take the form of the shorter AS1-S, some reads do extend to the postulated end of AS1-L at position ~5237. A number of long reads end before this position. **b** Mapping of the soft clipped reads with BLAT (http://genome.ucsc.edu/) reveal that many of these transcripts are undergoing splicing with the ELF2 transcript. The large number of polymorphisms observed in the mapped reads are due to the high error rate observed in Nanopore reads

Our approach to 3′RACE was slightly different from traditional methods as we used a primer in the first exon of AS1/2 to produce nearly full-length cDNAs, which were then processed into libraries for high throughput sequencing. Libraries were produced using total RNA from the cell lines YR2, L267 and FLK, three ovine and four bovine tumors, as well as six BLV infected sheep at 17 months post-inoculation. When mapped back to the BLV genome, the products of 3′RACE were mainly clustered in a region overlapping with BLV miR-B5-3p (Additional file 1: Fig. S2; Fig. 2). A canonical AAUAAA polyadenylation signal sequence (PAS) was found at position 6913–6918 immediately adjacent to the end of miR-B5-3p, however no GU rich consensus sequence was obvious (Fig. 2a). The observed poly A sequences were not confined to a single position but clustered at various points in a ~60 bp region between 6842 and 6910. This region also corresponds with a position where the antisense read coverage drops, indicating that the majority of BLV antisense transcripts utilize this poly A site, resulting in a short transcript (AS1-S) of approximately ~600 bp (Fig. 1; Additional file 1: Fig. S2). In all the samples, a number of reads were observed beyond this point, with a second cluster of poly A sequences observed around position 6171. No PAS was observed adjacent to this position and the presence of an adenine rich region overlapping with the poly A sequences suggests that this region served as the priming site for our oligo dT primer. A second canonical AAUAAA (PAS) was found at position 5257–5262 (again without an obvious GU rich consensus sequence). When 3′RACE was carried out with a primer adjacent to this PAS and YR2 as template, reads were observed 20 bp upstream (Fig. 2b). Reads showing evidence for this poly A tail were also observed in five of the libraries produced using a primer extending from the first AS exon. An additional 3′RACE library, using YR2 RNA as template, was sequenced on an Oxford Nanopore MinION. The long reads from this instrument showed a number of transcripts extending up to the region around position 5237 (Fig. 2d). The presence of a longer version of AS1 is also supported by PCR (Fig. 1b; Additional file 1: Fig. S1). Collectively these results indicate that a portion of the AS1 transcripts do extend into and beyond the BLV microRNA region, creating a long AS1 transcript (AS1-L). While some AS1-L transcripts appear to be polyadenylated at position 5237 the frequency appears low and in the MinION reads many of the AS1-L reads represent a host-viral fusion transcript, formed using canonical splice donor acceptor sites in the host and viral genomes (Fig. 2d). Additionally, it should be noted that the bovine tumor T1345 contains a large internal deletion that includes the PAS at position 5257–5262 (includes the last ~100 bp of AS1-L, assuming poly A tail at position ~5237) suggesting that at least in tumors, this portion of the transcript is dispensable.

In the case of AS2, the majority of the samples showed no evidence of a poly A tail, rather the AS2 transcript ended abruptly at position 2704. Closer examination of the sequences showed the transcript had also utilized canonical splice donor acceptor sites in the viral and host genomes to form a virus-host fusion transcript. In each tumor examined, the sequence forming the fusion with AS2 corresponded to the region of the host genome where the provirus was inserted (Fig. 2c). In the case of L267, in addition to BLV-host fusion transcripts, a small number of AS2 reads were observed with appetent poly A tails (Fig. 2c). No PAS was observed in the vicinity and the position of the poly A varied over a ~220 bp region with the majority slightly upstream of the splice site utilized in the formation of the BLV-host fusion transcript.

### BLV 3′LTR promoter activity and regulatory sequences affecting antisense transcription

**Fig. 3:**
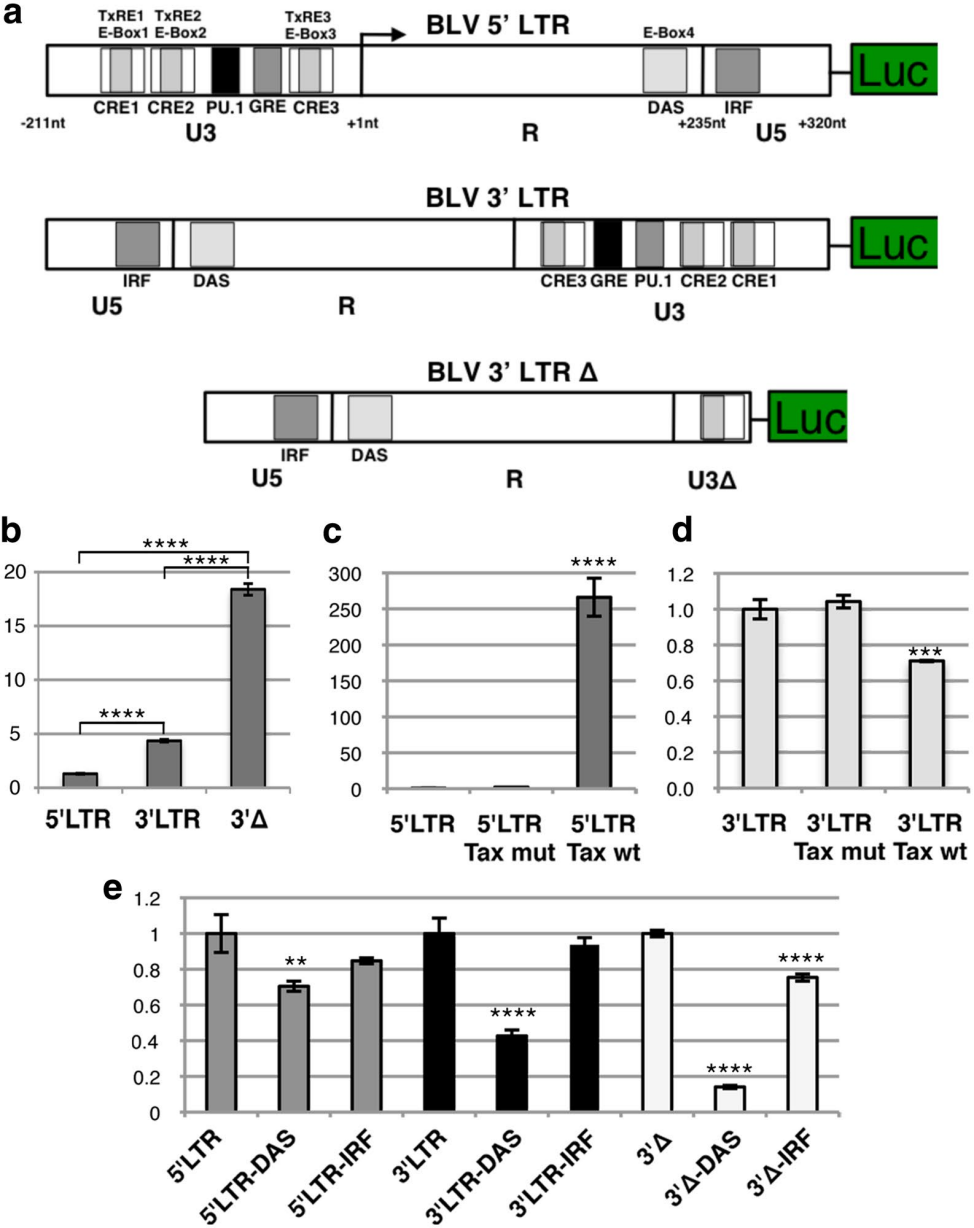
BLV 3′LTR antisense promoter activity and regulatory sequences affecting transcription. **a** Ideogram showing the three constructs used in luciferase assays, 5′LTR orientation, 3′LTR orientation and 3′LTR orientation with the majority of the U3 deleted (3′∆). Transcription factor binding sites from [24]. **b** Luciferase levels for the 5′LTR, 3′LTR and 3′∆ constructs. Comparison between the three constructs showed a significant difference among all three (p value <0.0001). **c** 5′LTR construct alone and co-transfected with a wild type (wt) Tax and transactivation deficient Tax (mut), which carries a E-to-K amino acid change at position 303 [39]. Tax wt caused a significant increase in luciferase levels (p value <0.0001). **d** Same as **c** but using the 3′LTR construct. Tax produced a significant (p value <0.001) drop in luciferase levels. **e** 5′LTR construct, mutation of IRF resulted in a modest but non significant drop in expression. Mutating DAS resulted in a statistically significant (p value <0.01) decrease (in line with previous reports). For 3′LTR, mutation in IRF caused modest, but non significant drop in expression, mutating DAS resulted in a significant drop (p value <0.0001). In the 3′∆ construct, mutating IRF and DAS caused a significant (p value <0.0001) decrease in luciferase levels (Luciferase levels are scaled to the left most construct in **b**, **c** and **d**. In E they were scaled to the appropriate wild type construct. Statistical significance was determined via Tukey’s multiple comparisons test, **p value <0.01; ***p value <0.001; ****p value <0.0001)

The capacity of BLV to drive sense transcription from the 5′LTR has been investigated in detail over the years, however the promoter activity of the 3′LTR remained unexplored. To confirm that the 3′LTR was capable of driving expression of an antisense transcript, we inserted the BLV LTR upstream of a luciferase construct in both the 5′ and 3′ orientations (Fig. 3a). In the 3′ orientation, luciferase activity was significantly higher than the basal activity seen in the 5′ orientation (Fig. 3a). The majority of regulatory motifs identified in the BLV 5′LTR are concentrated in the U3 region [17, 18]. Removal of the first 167 bp of U3 in the 3′LTR construct (3′∆) (Fig. 3a), resulted in significantly increased luciferase activity (Fig. 3b). As expected, expression of Tax, the potent viral transactivator, resulted in a dramatic increase of luciferase activity with the LTR in the 5′ orientation (Fig. 3c), while in the 3′ orientation Tax caused a significant reduction. It has previously been reported that there is an Interferon Regulatory Factor (IRF) binding site in U5 [17] and a E-Box in the R region (labeled as DAS: downstream activator sequence) [18]. To test if these motifs played a role in the 3′LTR antisense promoter activity, we carried out site directed mutagenesis. Disruption of the DAS motif caused a significant drop in luciferase activity for both the 5′LTR and 3′LTR constructs (Fig. 3e). In the case of the IRF motif, a modest although non significant reduction was seen in both constructs (Fig. 3e). Finally, disruption of the IRF and DAS motifs in the construct carrying the 167 bp deletion in U3 caused a significant reduction in luciferase activity for both motifs (Fig. 3e). Taken together these results show the 3′LTR is capable of driving antisense transcription in the BLV provirus in the absence of Tax and that the IRF and DAS motifs appear to play a role in both sense and antisense transcription.

### Coding potential

Having confirmed the promoter activity of the 3′LTR and estimated the approximate scope of the BLV antisense transcripts, we next examined them for coding potential using the Coding Potential Assessment Tool (CPAT) [19]. The limited number of annotated bovine long non-coding RNAs (lncRNAs) prevented development of a robust bovine specific model to calculate coding probability. As a result, human and mouse CPAT models were utilized. In addition to the BLV antisense transcripts, a number of known BLV and HTLV-1 protein coding genes and 220 bovine lncRNAs were analyzed. The known protein coding genes (including the HTLV-1 antisense transcript *HBZ*) were all assigned a high coding probability, while in contrast, >90 % of the bovine lncRNAs were assigned a coding probability less than 0.25 in both models (Additional file 1: Table S2). AS2 coding probability was low (0.003 human model, 0.022 mouse model), allowing us to classify it as an lncRNA. AS1-S/L were assigned an ambiguous coding probability of ~0.46 in the human and ~0.39 in the mouse model, with a potential open reading frame (ORF) of 264 bp (shared by both AS1-S and AS1-L). The small putative peptide sequence did not show any significant matches when used to search the non-redundant protein sequence (nr) database with BLASTP and the protein families database [20].

We next examined patterns of nucleotide variation in the region containing the putative peptide. Using our RNA-seq data, we extracted the sequence of the putative AS1-S/L ORF from each of our bovine and ovine tumors and supplemented these with six publicly available whole genome BLV sequences from NCBI. AS1-S/L ORF was characterized by a nucleotide diversity (average difference per nucleotide site for all pairwise comparisons of available sequences) of 0.0149. In comparison Tax, known to be essential, had a nucleotide diversity of 0.0106. Throughout the putative ORF, the majority of the nucleotide diversity of AS1-S/L, like that of Tax, was concentrated in the less constrained 3rd codon position of the negative strand (0.034 and 0.0247 for AS1-S/L and Tax respectively) (Additional file 1: Fig. S3; Additional file 1: Table S3). It should be noted though, that for just over 2/3 of the sequence, the potential AS1-S/L ORF overlaps with parts of the R3 and G4 ORFs on the sense strand. Additionally, the 3rd codon position of G4 on the sense corresponds to the 3rd codon position of the potential AS1-S/L ORF on the negative strand. Nevertheless, this pattern of nucleotide diversity was not confined to the overlapping sequences and extends into the non overlapping region (no overlap 0.0236 vs no overlap 3rd 0.0521; Additional file 1: Fig. S3; Additional file 1: Table S3). Consequently, the evidence for coding potential in AS1-S/L is suggestive, but not definitive, making it best to classify AS1-S/L as transcripts of unknown coding potential (TUCP) [21].

### Sub-cellular localization of BLV antisense transcripts

**Fig. 4:**
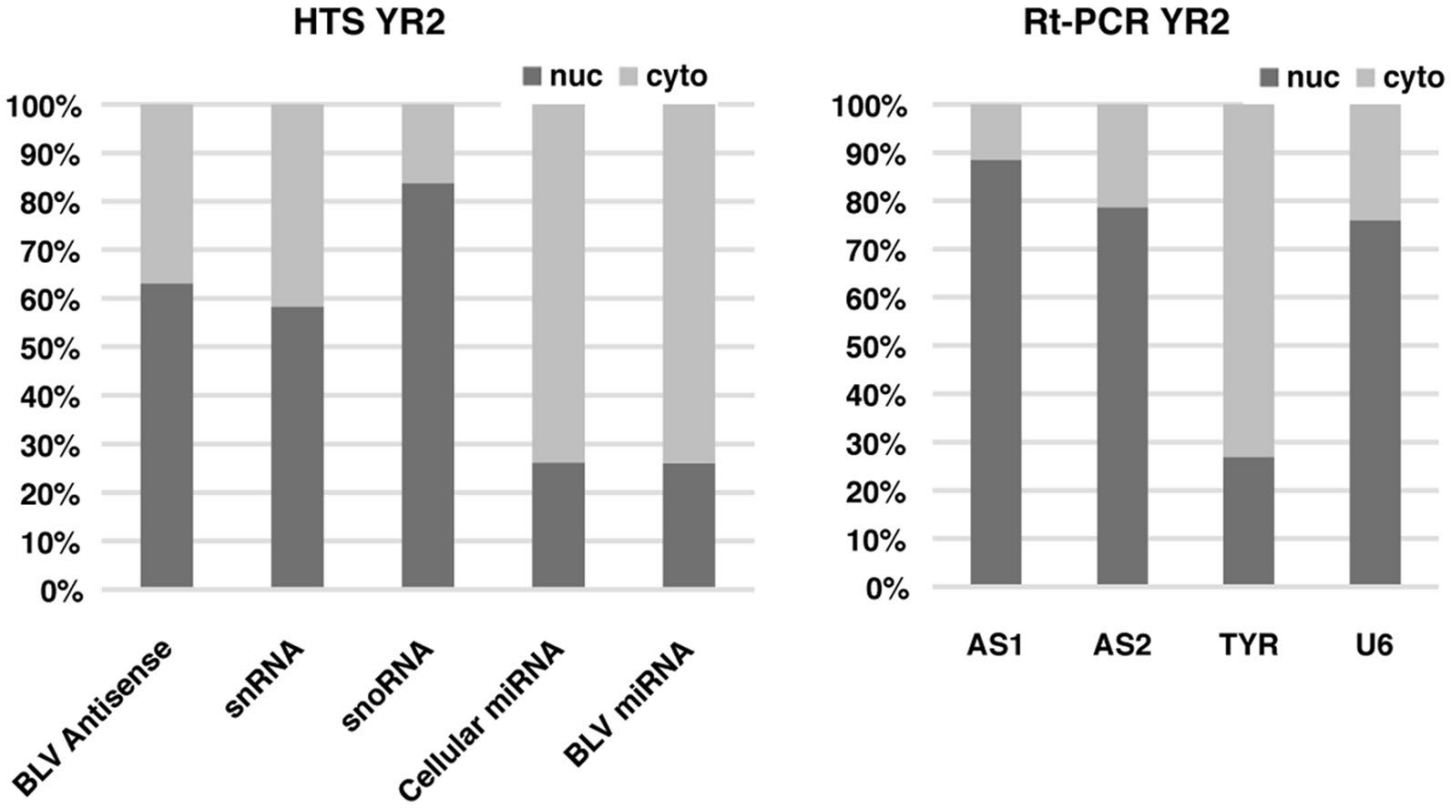
Sub-cellular localization of BLV antisense transcripts. YR2 was fractionated into cytoplasmic and nuclear enriched pools of RNA, followed by production of total RNA and small RNA libraries for high throughput sequencing (HTS). Read count for each library was normalised and enrichment in the cytoplasm/nucleus calculated for the different classes of transcript. The HTS results were confirmed with RT-PCR using primers for the antisense BLV transcripts AS1 and AS2. Primers for TYR (cytoplasmic) and U6 (nuclear) were used to confirm fractionation efficacy. Both approaches confirm that the BLV antisense transcripts has a primarily nuclear localization (*snoRNA* small nucleolar RNA, *snRNA* small nuclear RNA)

Given the ambiguous coding potential of the BLV antisense transcripts we next sought to determine their location in the cell to better understand their potential functions. This was achieved by carrying out high throughput sequencing of total and small RNA libraries using cytoplasmic and nuclear enriched RNA fractions from the YR2 cell line. The resultant small RNA libraries confirmed that the BLV microRNAs (like the cellular microRNAs) were enriched in the cytoplasmic fraction (Fig. 4). In the case of the total RNA libraries, the BLV antisense reads (all antisense reads were considered together) were enriched in the nuclear fraction (Fig. 4). Absolute quantification via real-time PCR using primers spanning the splice site of AS1-S/L and AS2 also demonstrated that both transcripts were enriched in the nuclear RNA fraction (Fig. 4).

### BLV microRNA guided cleavage of AS1-L

**Fig. 5:**
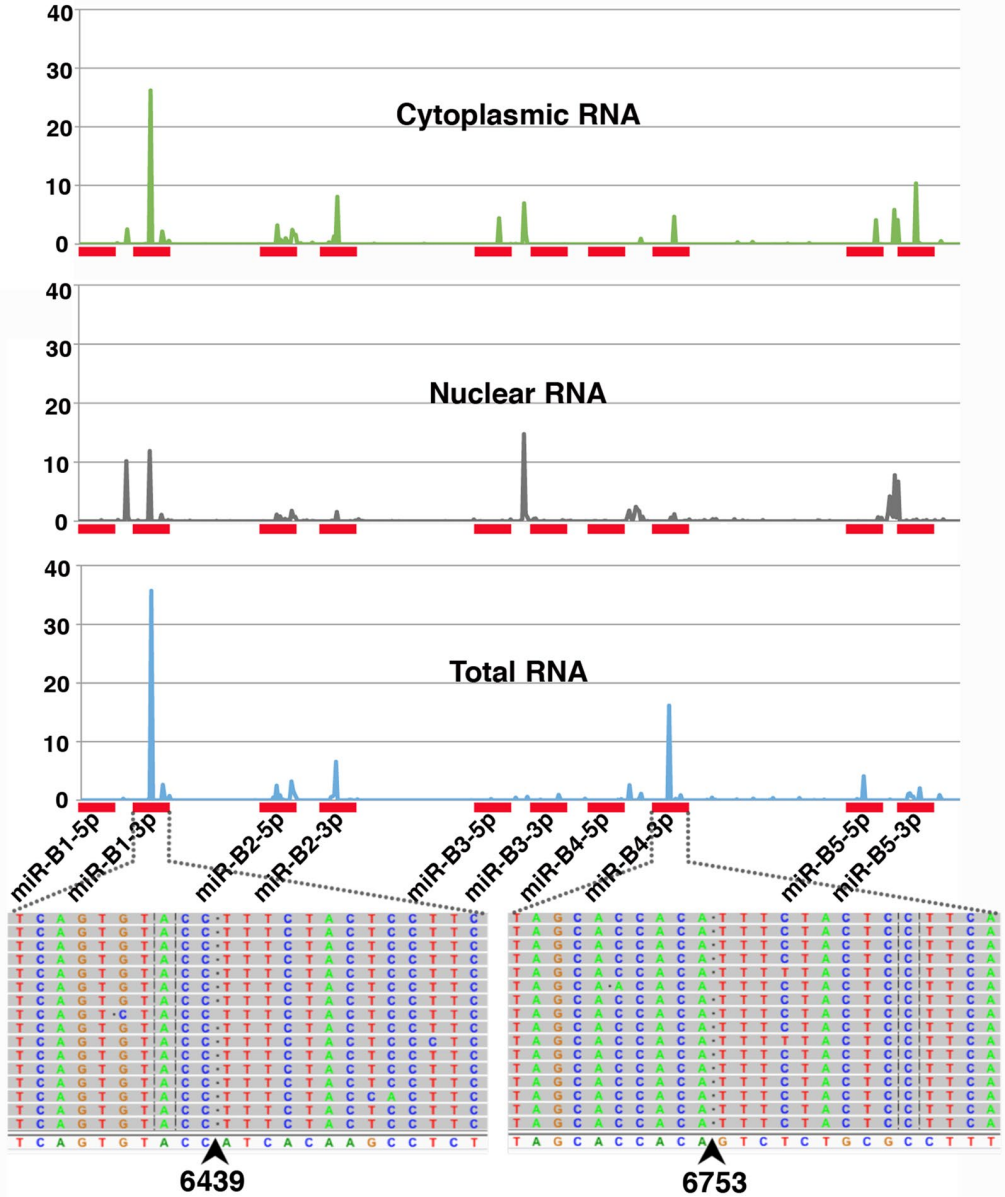
BLV microRNA mediated cleavage of the AS1-L transcript. Shown on the *X axis* is the region of the BLV genome containing the BLV microRNAs (denoted by *red rectangles*). *Graphed* above is the percentage of high throughput sequencing reads showing evidence of cleavage at each base position. Libraries were prepared from total YR2 RNA and YR2 cells that were fractionated into cytoplasmic and nuclear enriched fractions. Below the *X axis* is shown the precise base pair position of cleavage for the peaks observed at miR-B1-3p and miR-B4-3p. The reference sequence is shown at the bottom and the individual reads above. The first 10 bp of the reads match the reference sequence, the sequence to the right of the cleavage point (*arrow*) corresponds to the RNA oligo ligated to the free 5′ end. Sites of cleavage were enriched between bp 10 and 11 of a subset of the mature microRNAs. The frequency of reads showing evidence of cleavage was increased in the cytoplasmic fraction. Free 5′ ends observed outside bp 10 and 11 of the microRNA in each sample are likely the product of RNA degradation

The majority of the AS1 transcripts end adjacent to the end of miR-B5-3p (AS1-S), however some ~19 % extended beyond this point (Figs. 1, 2; Additional file 1: Fig. S2) and overlap the BLV microRNAs. As a result these AS1-L transcripts should be sliced via the action of RISC (RNA-induced silencing complex), with cleavage occurring between the 10th and 11th base pair position of the mature microRNA [22]. To identify the products of cleavage, a modified version of RNA-ligase-mediated (RLM) 5′RACE [22] was employed and combined with high throughput sequencing. Using RNA from the YR2 cell line, we found that ~66 % of the reads mapping to the ~550 bp BLV microRNA region had a 5′ end that fell between the 10th and 11th base pair of one of the BLV microRNAs. The majority of the cleavage appears to be driven by five of the BLV microRNAs. Of these, miR-B1-3p was responsible for 35.7 % of the reads, miR-B2-5p 3.4 %, miR-B2-3p 7.5 %, miR-B4-3p 16.2 % and miR-B5-3p 2.9 % (Fig. 5; Additional file 1: Table S1). We also produced a library using total RNA extracted from the PBMCs of a sheep 19 months post-infection and found that ~26 % of the reads mapping to the BLV microRNA region showed evidence of cleavage mediated by the BLV microRNAs (Additional file 1: Table S1). To examine if this cleavage was occurring in the cytoplasm or nucleus, we produced nuclear and cytoplasmic enriched RNA pools from YR2 and carried out the same modified 5′RACE and sequencing. Reads showing evidence of cleavage mediated by the BLV microRNAs were higher in the cytoplasmic (~54 %) versus the nuclear fraction (17 %), indicating RISC mediated cleavage in the cytoplasm (Fig. 5; Additional file 1: Table S1). The percentage of reads mapping to the BLV microRNA region was similar within each fraction (~56 % nuclear, ~57 % cytoplasmic).

### Altering the microRNA region in the BLV provirus reveals transcriptional interactions between the antisense transcripts and viral microRNAs

**Fig. 6:**
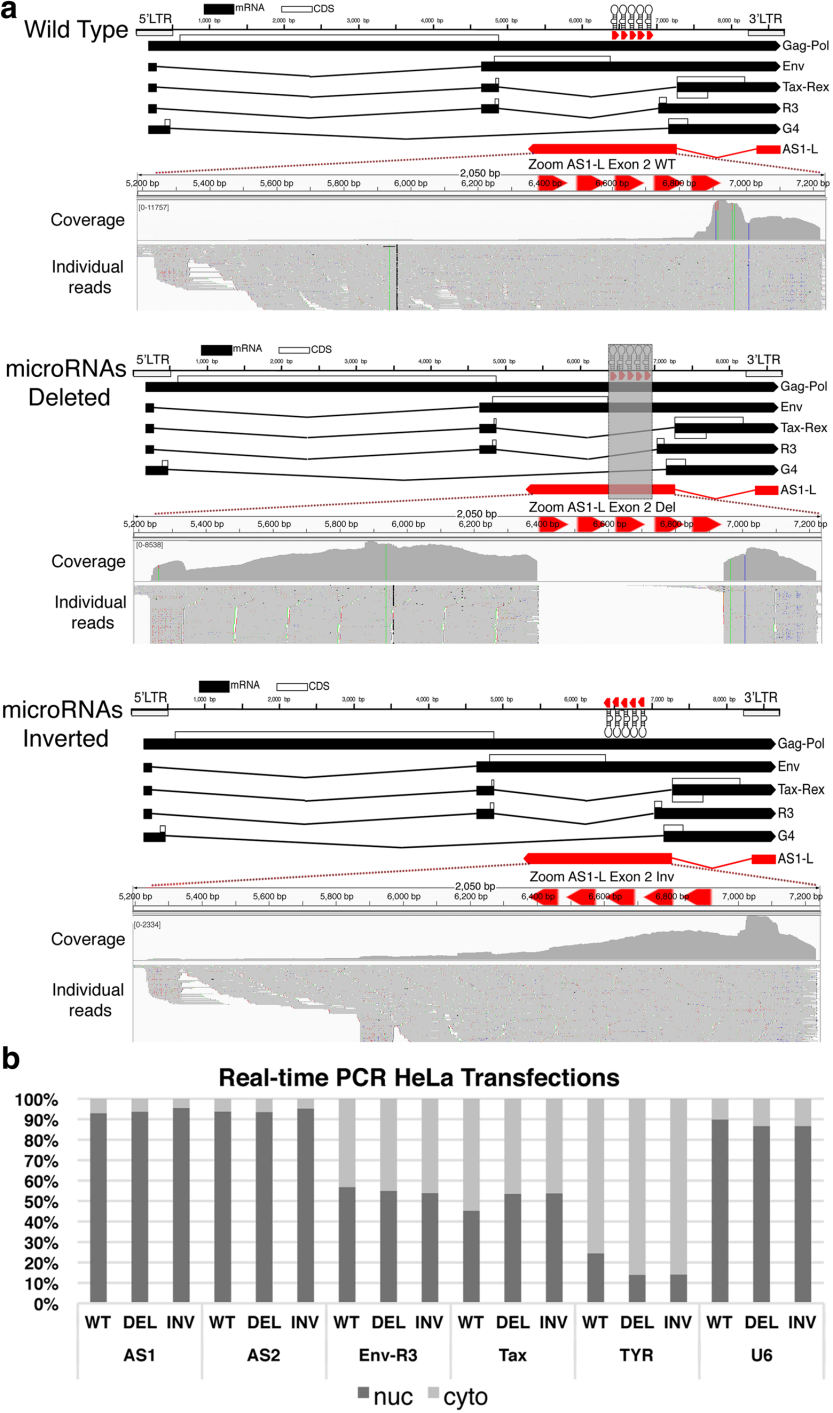
Mutant proviruses altered in the microRNA region reveal interactions between the antisense transcripts and the viral microRNAs. **a** Ideograms showing the three BLV proviruses used in transfections, the first a wild-type, the second where the microRNAs were deleted (*grey box* indicates the region removed), the third where the BLV microRNAs were inverted. 3′RACE on total RNA from HeLa cells transfected with the three constructs shows that the microRNA deletion/inversion increases the fraction of AS1-L reads. In the case of the wild-type provirus and the microRNA-deleted provirus, reads were mapped to the wild type provirus sequence. In the case of the microRNA-inverted provirus, they were mapped to a proviral genome with microRNAs inverted to match the provirus used. **b** Nuclear versus cytoplasmic enrichment for BLV sense and antisense transcripts obtained via real-time RT-PCR with nuclear and cytoplasmic enriched RNA from HeLa cells transfected with either the wild type BLV pBLV344 provirus, the provirus with the BLV microRNAs deleted or with the orientation of the BLV microRNAs inverted. Removing or inverting the BLV microRNAs had no effect on the localization of the sense or antisense transcripts. *TYR* cytoplasmic enriched control, *U6* nuclear enriched control

Given the observed cleavage of AS1-L in the cytoplasm we next sought to examine if the absence of BLV microRNAs would have any effect on the relative fraction of AS1-S/L produced. To achieve this, we designed two altered BLV proviruses. In the first mutant, the BLV microRNAs were deleted, while in the second they were inverted to prevent cleavage of AS1-L (in both cases the PAS just upstream of BLV miR-B5-3p was also removed (Fig. 6a). These proviruses were transfected into HeLa cells, followed by 3′RACE and high throughput sequencing. Like the situation in tumors, the WT provirus 3′RACE library showed a rapid drop off in coverage in the region containing the BLV microRNAs (Fig. 6a). In contrast, for the microRNA deleted provirus the level of coverage remained relatively consistent across the length of AS1-L, while in the inverted provirus, there was a gradual decrease in coverage along the length of AS1-L (Fig. 6a). As a result, deletion or inversion of the BLV microRNAs and the adjacent PAS appears to increase the fraction of AS1-L transcripts present.

The increased fraction of AS1-L transcripts could be explained in two ways. Firstly, the RNA pol III positioned at the BLV microRNAs may inhibit RNA pol II progression allowing the PAS adjacent to BLV miR-B5-3p microRNA to mediate polyadenylation of the antisense transcripts, thereby forming AS1-S. By removing these elements, AS1-L levels increase. Secondly, by removing the microRNAs we prevent the cleavage of AS1-L in the cytoplasm, increasing the apparent number of AS1-L reads and causing their accumulation in the cytoplasm. In an attempt to distinguish between these two possibilities, we fractionated transfected cells in into nuclear and cytoplasm enriched RNA pools. Real-time PCR with primers spanning the splice sites for AS1-S/L, AS2 and BLV sense transcripts did not show differences in the sub-cellular distribution of transcripts for the three proviruses (Fig. 6b). As a result, it does not appear that the increased fraction of AS1-L transcripts observed is a consequence of eliminating BLV microRNA mediated slicing of AS1-L, rather it suggests the first scenario involving RNA pol II and pol III interference.

### Knock down of AS1 with locked nucleic acids (LNAs)

A mix of three LNAs, targeting different parts of AS1 were used to treat both the YR2 and L267cell lines, with a biological replicate for each cell line, at concentrations of 5 and 10 μM. In addition, the cell lines were treated with 10 μM of control LNAs and with a mock treatment of H_2_O. Dunn’s multiple comparisons test was then used to examine differences between the individual samples and found that at the 10 μM concentration, the drop (>50 %) between the control and AS specific LNAs was significant (p value = 0.0347). In the case of AS2 a significant difference was observed between the treatments (Kruskal–Wallis test, p value = 0.0208), however Dunn’s multiple comparisons test failed to show any difference between individual samples (Additional file 1: Fig. S4A). The real time assay for the BLV sense transcript Tax failed to show amplification or was indistinguishable from the background in all the YR2 and L267 treatments (Tax expressing cells showed robust amplification with an average Ct of 21.5). As a result, it appears that knock down of AS1 does not cause a detectable reactivation of transcription from the 5′LTR. Finally, there was no apparent difference in the viable cell counts in the treated and control cells at the end of the experiment (Additional file 1: Fig. S4B).

## Discussion

It has been known for over a decade that BLV’s close relative HTLV-1 encodes an antisense transcript [10] and like HTLV-1, the BLV 3′LTR remains unmethylated in tumors [16], begging the question of a BLV antisense transcript. Prior to the adoption of RNA-seq, identifying these transcripts required a certain amount of luck in the placement of primers/probes for Rt-PCR/Northern blotting. However, as can be seen from Fig. 1 the use of RNA-seq, especially in combination with stranded libraries, made the presence of antisense reads in BLV tumors obvious. The consistent pattern of antisense transcription across all the BLV samples sequenced to date strongly suggests that these transcripts fulfill an essential function in the biology of BLV and leukemogenesis. Furthermore, in the majority of samples little or no evidence of sense transcription was observed, mirroring results recently obtained in HTLV-1 [9]. While we saw evidence of antisense without sense transcription, we did not see the inverse. RNA-seq of YR2_LTaxSN_/L267_LTaxSN_ and the 3′RACE library from the FLK cell line showed that antisense expression levels and splicing patterns were comparable to that seen in ovine and bovine tumors (Additional file 1: Table S1; Additional file 1: Fig. S2), despite robust sense transcription. A related question was the temporal pattern of expression of the BLV antisense transcripts. By combining stranded RNA-seq, high-throughput mapping of proviral integration sites, 3′RACE and small RNA-seq of PBMCs from asymptomatic BLV infected sheep (Additional file 1: Table S1) we could be confident that antisense/microRNA transcription is a feature of BLV infected B cell clones present at the polyclonal stage and not confined to the tumor clone. Given the tractability of the BLV sheep model, in comparison to the difficulty of studying primary HTLV-1 infections [23] it will be interesting to extend this analysis back to the earliest stages of infection to examine the dynamics of sense, antisense and microRNA expression from BLV. The Tax protein has been seen as the primary actor in both HTLV-1 and BLV, however over the course of infection it is now apparent that its expression is the exception rather than the rule, while antisense transcription appears the rule. Probing the initial infection in experimentally infected sheep may elucidate if and how Tax sets the stage for tumor development and if antisense transcription displays more dynamism in comparison to its consistent expression in both the established nonmalignant and expanded tumor clones.

The presence of BLV antisense transcription implies that the BLV LTR (like that of HTLV-1) is capable of driving transcription in both directions. Extensive work over the years has revealed a rich set of regulatory motifs bound by cellular transcription factors and Tax [18, 24], regulating the BLV 5′LTR. As expected given the evidence of antisense transcription, we found that the BLV LTR was able to drive transcription in the orientation of the 3′LTR and with a significantly higher activity compared to the weak basal activity typical of the 5′LTR. Our data are consistent with observations reported by Van Driessche et al. [25] demonstrating RNA Pol II recruitment to the 3′LTR-host boundary and its association with positive epigenetic marks. Removal of the majority of the regulatory motifs in U3, taken with the results from Tax co-transfection suggest that the motifs in U3 do not have a role in driving, but rather obstruct BLV antisense transcription. In contrast, the motifs in the R and U5 regions appear to have a dual role in driving expression from both the 5′ and 3′LTRs. Taken in conjunction with our sequencing results it appears that consistent antisense expression from the 3′LTR is a feature of the BLV provirus in both nonmalignant and tumor clones. In contrast, the 5′LTR is tightly regulated and does not show evidence of consistent expression. As a consequence, it is apparent that Tax is not required for maintaining malignancy and the absence of this immuno-dominant viral protein in transformed cells plays a vital role in BLVs immune evasion strategy. In contrast, the permanent production of both antisense transcripts and microRNAs, besides pointing to an essential function in tumorigenesis, indicates that these transcripts are incapable of eliciting an effective host immune response.

Having established the presence of BLV antisense transcripts we attempted to determine their approximate size with a combination of short and long reads. It was somewhat surprising to see that the majority of AS1 is only ~600 bp in length, a much shorter transcript than *HBZ* (>2 kb) in HTLV-1 [7], while AS2 exon 2 was small (~125 bp) and generally forms fusion transcripts with the host genome. Our 3′RACE and long read sequencing showed that the longer version of the AS1 transcript (AS1-L > 2 kb) could extend to a poly A tail just beyond the canonical AAUAAA (PAS) at 5257–5262. However the balance between the use of this poly A tail and the formation of fusions with the host genome remains unknown and will likely depend on the location of the provirus in the genome. The use of long read technologies such as the MinION will be particularly useful in answering this question, as full-length sequences can unambiguously determine the structure of fusion transcripts.

RNA-seq data indicated that the majority of the AS1 reads take the form of the AS1-S variant, which terminates next to the end of miR-B5-3p and is considerably smaller than *HBZ*. In the case of the AS1-L, there is an overlap with the BLV microRNAs leading to the cleavage of AS1-L via action of the RISC complex [26]. Our modified 5′RACE experiments confirmed this and showed that RISC mediated cleavage predominated in the cytoplasm (the fraction also enriched for BLV microRNAs in YR2). The limited number of microRNAs involved in cleavage is intriguing as previous work indicates that all the BLV microRNAs tested associate with the RISC complex [13]. This may point to secondary structures in BLV AS1-L that inhibit cutting at certain positions, however the significance of this observation remains to be determined and more samples are required to see if this pattern is consistent. Another question requiring future work is the fraction of reads that are subjected to cleavage. The majority of the AS1 transcripts (AS1-S) do not contain the region complementary to the BLV microRNAs. Additionally, a majority of the antisense transcripts are retained in the nucleus while the BLV microRNAs are predominantly found in the cytoplasm. How the nuclear versus cytoplasmic localization of AS1-L and the balance between AS1-S versus AS1-L evolves over the entire life cycle of the virus, in different cell types and in times of cellular stress remains an open question. Exploring these dynamics could be important to understand the significance of microRNA mediated cleavage of AS1-L.

Other microRNA producing viruses such as SV40 [27] and Epstein–Barr virus [28] possess viral microRNAs that cleave products from the complementary strand. It is thought that this microRNA mediated regulation manages the transition from latency (low stress) to the lytic stage (high stress). With stress mediated suppression of microRNA production, cleavage of the antisense product is relaxed, allowing it to signal activation of the latent virus [29]. It is conceivable that a similar mechanism is involved in regulating the relative abundance of the BLV AS1-S/L transcripts as RNA pol III transcription is known to be regulated by cell stress and cell cycle stage [30]. In addition to reducing the amount of microRNAs available for cleaving the AS1-L, changes in RNA pol III promoter occupancy and transcription could increase the fraction of AS1-L produced by removing RNA pol III complexes on the sense strand potentially impeding RNA Pol II and encouraging production of the larger transcript (AS1-L). It is apparent in Fig. 1 and Additional file 1: Fig. S2 that antisense coverage diminishes as we enter the region containing the BLV microRNAs. RACE experiments with RNA from HeLa transfections showed that deleting/inverting the microRNAs/PAS in the BLV provirus results in a higher fraction of AS1-L (Fig. 6a) without altering the fraction of AS transcripts localized in the nucleus or cytoplasm. This suggests the removal of an insulator that prevents progression of RNA pol II rather than reduced microRNA mediated cleavage of AS1-L. Our data are in agreement with recent Chip-seq work by Van Driessche et al. [25] showing evidence of a high RNA pol III occupancy peak in the BLV microRNA region with a large RNA pol II occupancy peak just downstream of this position, consistent with RNA pol III–RNA pol II collision.

The significant size difference between AS1-S and AS1-L is intriguing as both contain the same potential ORF. Assuming that the ORF does produce a functional protein, its potential role remains opaque as database searches did not reveal any significant homology with known protein domains. In HTLV-1, a number of roles have been assigned to the HBZ protein, including the suppression of HTLV-1 5′LTR transcription and repression of the host immune response [7]. In HTLV-2 the antisense protein APH-2 also plays a role in suppressing viral transcription [31]. However, the potential protein in BLV AS1-S/L (87 amino acids) is considerably smaller than that reported for HTLV-1 (206 amino acids spliced/209 amino acids unspliced) and HTLV-2 (183 amino acids) which argues against an exactly analogous protein (although the final effect may be equivalent). In addition to the roles ascribed to the HBZ protein, it has been reported that the *HBZ* mRNA is capable of supporting proliferation [12] and is primarily retained in the nucleus [32], suggesting an lncRNA like function. Only a small portion of AS1-L is occupied by the potential ORF and the AS1-L\S transcript is primarily retained in the nucleus, again hinting at a potential lncRNA like role. Recently, there has been a rapid expansion of our understanding of the numerous roles lncRNAs play in the cell [33]. Many of these lncRNAs also show a predominately nuclear localization and are involved in transcriptional regulation [34]. Additionally, many transcripts previously described as lncRNAs possess small ORFs (<100 amino acids) that do not resemble those found in well characterized mRNAs [35]. The situation for the BLV antisense transcripts appears similar. Thus, it is reasonable to propose that one role of the BLV AS1-S/L may be silencing of the 5′LTR. Future work using in vitro infection with altered proviruses will be needed to firstly establish the precise role AS1-S/L plays in the life cycle of BLV and secondarily if these different roles are carried out via the RNA or potential protein product. With this information in hand it will be interesting to compare and contrast the roles AS1-S/L and *HBZ* transcript play and given the tractability of the BLV model, make targeted changes to the transcript and examine the effect on tumor development.

Looking beyond AS1-S/L there remains the enigma of the AS2 transcript, where at the moment the potential functions are even more obscure than for AS1-S/L. No comparable antisense transcript has been reported for HTLV-1 and no potential ORF is present in the transcript. Furthermore, the expression levels of AS2 are lower than that observed for AS1 (Fig. 1; Additional file 1: Fig. S2). Additionally, the second exon of AS2 is small (125 bp) creating a transcript containing just over ~400 bp of sequence from the BLV genome. However, the consistent splicing of AS2 with the host genome results in the production of a fusion transcript containing ~400 bp from BLV and additional sequences from the host genome. As a result the AS2 transcript size and composition will differ from cell to cell depending on provirus location in the host genome. It has recently been reported for HTLV-1 that antisense transcripts can also form fusion transcripts with the host genome [9]. Additionally we also see extensive splicing between the common AS exon 1 and the host genome (using canonical splice acceptor sites) and find evidence that these fusions may play a role in leukemogenesis. These observations are outlined in detail in Rosewick et al. submitted and point to an important role for antisense dependent viral/host transcripts in the biology of deltaretroviruses.

A striking feature of many HTLV-1 and BLV proviruses is the large deletions frequently observed in tumors, that can remove over half the proviral DNA, often including the entre 5′LTR and many vital ORFs, while always retaining the 3′LTR [9, 13]. These observations strongly indicate that *HBZ* plays an important role in leukemogenesis and a similar pattern of deletions in BLV may point to an analogous role for the BLV AS1-S/L. The case of the BLV provirus T1345 is intriguing for this reason as the large deletion gives us a clue about which BLV antisense transcripts are important in the tumor. In this provirus a ~4.3 kb deletion removes many essential viral genes but retains the BLV microRNAs [13] and continues to display robust antisense expression (Additional file 1: Fig. S5). This deletion also removes the second exon of AS2 and the last ~100 bp of AS1-L indicating that these transcripts/regions are dispensable in the tumor.

Our attempt to investigate the function of the AS1-S/L transcript via knock down was hampered as the YR2 and L267 B cell lines are refractory to transfection and require high levels of LNAs for unassisted uptake, making optimization and long-time courses cost prohibitive. Consequently, we carried out a limited number of assays and designed our experiment to examine if knock down of AS1-S/L could lead to the re-activation of viral transcription from the 5′LTR. It has been known for some time that the lack of 5′LTR based expression is imposed via histone repressive marks and DNA methylation at the 5′LTR [15, 16] and we hypothesized that the AS1-S/L has a role in maintaining this state. While we did achieve a significant knock down of AS1-S/L we did not see any evidence of transcription from the 5′LTR and based on final cell counts there was no apparent effect on cell viability (Additional file 1: Fig. S4). More prolonged or complete knock down may be required to produce an effect, however given the large quantities of LNAs required for unassisted uptake this was not practical. Previous work by Colin et al. [15] showed that following experimentally induced chromatin remodeling in the YR2 cell line there is a rapid return to a closed chromatin conformation. The AS-1S/L transcripts may play a role in this process and in order to observe any effect on 5′LTR based expression, AS1-S/L knock down must be coupled with prior relaxing of chromatin. It will be interesting to examine if combining LNA knock down of AS1 and treatment to open chromatin changes the patterns of epigenetic marks observed at the BLV 5′LTR.

## Conclusions

The identification of BLV antisense transcripts taken in conjunction with the recent identification of the BLV microRNAs [13, 14] represent a major shift in our understanding of BLV pathogenesis. In contrast to the prevailing paradigm of a silent provirus, our work reveals that the BLV provirus is a prodigious producer of viral microRNAs and constitutively expresses antisense transcripts in all tumors examined. The consistent expression of these transcripts in both leukemic and nonmalignant clones points to a vital role in the life cycle of the virus and its tumorigenic potential. Additionally, the cleavage of the AS1-L transcript by the BLV encoded microRNAs and the transcriptional interference between the two viral RNA species suggest a shared role in the regulation of BLV. While additional work is required to elucidate the precise roles of the BLV antisense transcripts, it is hoped that the greater tractability of the BLV model will provide key insights into HTLV-1 induced leukemia and provide a test bed for exploring potential treatment options.

## Methods

See Additional file 1: Text S1 for full details of materials and methods used.

### Ovine/bovine samples and cell lines

Primary ovine and bovine tumors were part of an existing BLV induced tumor collection maintained at −80 °C and described previously [13]. Asymptomatic sheep samples came from animals infected with the molecular clone pBLV344 following experimental procedures approved by the University of Saskatchewan Animal Care Committee, following Canadian Council on Animal Care Guidelines (Protocol #19940212). The cell lines YR2 and L267 were originally derived from ovine B-cell tumors, do not express viral mRNAs originating in the 5′LTR [13], L267_LtaxSN_ and YR2_LtaxSN_ were produced via transduction with the pLTaxSn retroviral vector expressing Tax [13].

### RNA sequencing

The Illumina TruSeq Total RNA stranded kit was used to prepare ribosomal RNA depleted libraries followed by sequencing on an Illumina HiSeq 2000 (2 × 100 bp paired-end reads), producing approximately 60 million raw paired-end reads per library. For small RNAs the Illumina TruSeq Small RNA Library Preparation Kit was used followed by sequenced on an Illumina NextSeq 500 (75 bp single reads). Reads were mapped to appropriate bovine UMD3.1 or ovine OAR3.1 genomes as well as BLV YR2 provirus genome (NCBI Accession: KT122858) using STAR (v2.3.1.u) and BWA (See Additional file 1: Text S1).

### End point PCR

SuperScript III Reverse Transcriptase (Life technologies) was used to produce cDNA. PCR primers used are listed in Additional file 1: Table S4.

### High-throughput sequencing of BLV integration sites

The Bioruptor Pico (Diagenode) was used to shear 5ug of DNA, followed by indexing with Nextera XT indexes and sequencing on a Illumina MiSeq instrument. Reads were mapped to the host-provirus hybrid genome with BWA and numbers were determined for each proviral integration site using in-house R and Perl scripts.

### Proviral load quantification

Proviral load was assessed with PrimeTime qPCR Assays (IDT) using diluted DNA from the YR2 cell line to produce a standard curve. Primers used are listed in Additional file 1: Table S4.

### Identification of 5′ and 3′ ends with RACE

The GeneRacer Kit (Life Technologies) was used for 5′ and 3′RACE followed by high throughput sequencing of the resultant PCR products. Near full-length cDNA transcripts were also produced by PCR followed by either shearing with a Bioruptor Pico (Diagenode) and sequencing on a MiSeq instrument or sequencing of the full-length transcripts on a MinION instrument (Oxford Nanopore Technologies).

### Luciferase assays

The LTR from BLV was derived from the pBLV344 plasmid [36], inserted into the pGL3 basic luciferase reporter plasmid. Constructs were transfected in triplicate with Lipofectamine 2000 (Life technologies) and processed using the Dual-Glo Luciferase Assay System (Promega). Statistical significance was assessed with Tukey’s multiple comparisons test, a p value <0.05 considered statistically significant.

### Protein coding potential and nucleotide diversity

The Coding-Potential Assessment Tool (CPAT) [19] was used to assess the coding potential of the BLV antisense transcripts. Sequence conservation was examined using sequences extracted from the bovine and ovine tumors sequenced as well as six genomes from NCBI (http://www.ncbi.nlm.nih.gov/nuccore), using the same approach outlined in Rosewick et al. [13].

### Sub-cellular location of BLV antisense transcripts

Cytoplasmic and nuclear fractions were produced from YR2 following the method outlined in Weil et al. [37]. RNA sequencing was carried out as outlined above. Real time PCR based absolute quantification was carried out on a ABI Prism 7900HT Sequence Detector System (Applied Biosystems) using ABsolute Blue QPCR SYBR Green ROX Mix (Thermo Scientific) and the appropriate PCR product in a plasmid to produce a standard curve.

### Identifying products of RISC mediated cleavage

The products of cleavage were identified with the protocol of Davis et al. [22] with the addition of high throughput sequencing of the resultant PCR products on a Illumina MiSeq. The precise position of cleavage was determined via mapping to the BLV provirus genome with BWA and a custom BASH script.

### Deleting and inverting the BLV microRNAs

The altered proviruses used the wild type molecular clone pBLV344 [36] as the starting material. In the deleted provirus 554 bp containing the BLV microRNAs was removed and replaced with the MluI restriction site. In the inverted provirus the BLV microRNAs were reinserted into this MluI restriction site in an inverted orientation. The constructs were transfected into HeLa cells with Lipofectamine 2000 (Life technologies) and real time PCR and 3′RACE carried out as described above.

### Antisense knock down with locked nucleic acid antisense oligos (LNAs)

The antisense transcript was targeted with a mix of three locked nucleic acid antisense oligos (Exiqon) introduced via unassisted uptake [38] in duplicate for a half million YR2 and L267 cells. Final concentrations of 5 and 10 μM were tested, as well as the LNA longRNA GapmeR Negative control A (Exiqon), at a final concentration of 10 μM and a mock treatment of H_2_O. Real time PCR was carried out as outlined above and statistical significance assessed via a Kruskal–Wallis test and Dunn’s multiple comparisons test, p values of ≤0.05 were considered to be statistically significant.

## Additional file

10.1186/s12977-016-0267-8 RT-PCR in bovine and ovine tumors. **Figure S2**. Eighteen 3’RACE libraries mapped to the BLV genome. **Figure S3**. BLV AS1 potential open reading frame. **Figure S4**. Knock down of AS1 with locked nucleic acids. **Figure S5**. Continued antisense transcription in the defective provirus T1345. **Table S1**. RNA sequencing results. **Table S2**. Output of Coding Potential Assessment Tool (CPAT). **Table S3**. Sequence conservation of BLV AS1 and Tax sequences. **Table S4**. Oligonucleotides used. **Text S1**. Extended materials and methods.
