# Supplementary material for: Characterization of novel Bovine Leukemia Virus (BLV) antisense transcripts by deep sequencing reveals constitutive expression in tumors and transcriptional interaction with viral microRNAs
Source: Retrovirology. 2016 May 3;13:33. doi: 10.1186/s12977-016-0267-8 (PMC4855707; doi:10.1186/s12977-016-0267-8)
Supplement: Supplementary file 1 — 10.1186/s12977-016-0267-8 RT-PCR in bovine and ovine tumors. Figure S2. Eighteen 3’RACE libraries mapped to the BLV genome. Figure S3. BLV AS1 potential open reading frame. Figure S4. Knock down of AS1 with locked nucleic acids. Figure S5. Continued antisense transcription in the defective provirus T1345. Table S1. RNA sequencing results. Table S2. Output of Coding Potential Assessment Tool (CPAT). Table S3. Sequence conservation of BLV AS1 and Tax sequences. Table S4. Oligonucleotides used. Text S1. Extended materials and methods. [file 12977_2016_267_MOESM1_ESM.pdf]

Figure S1

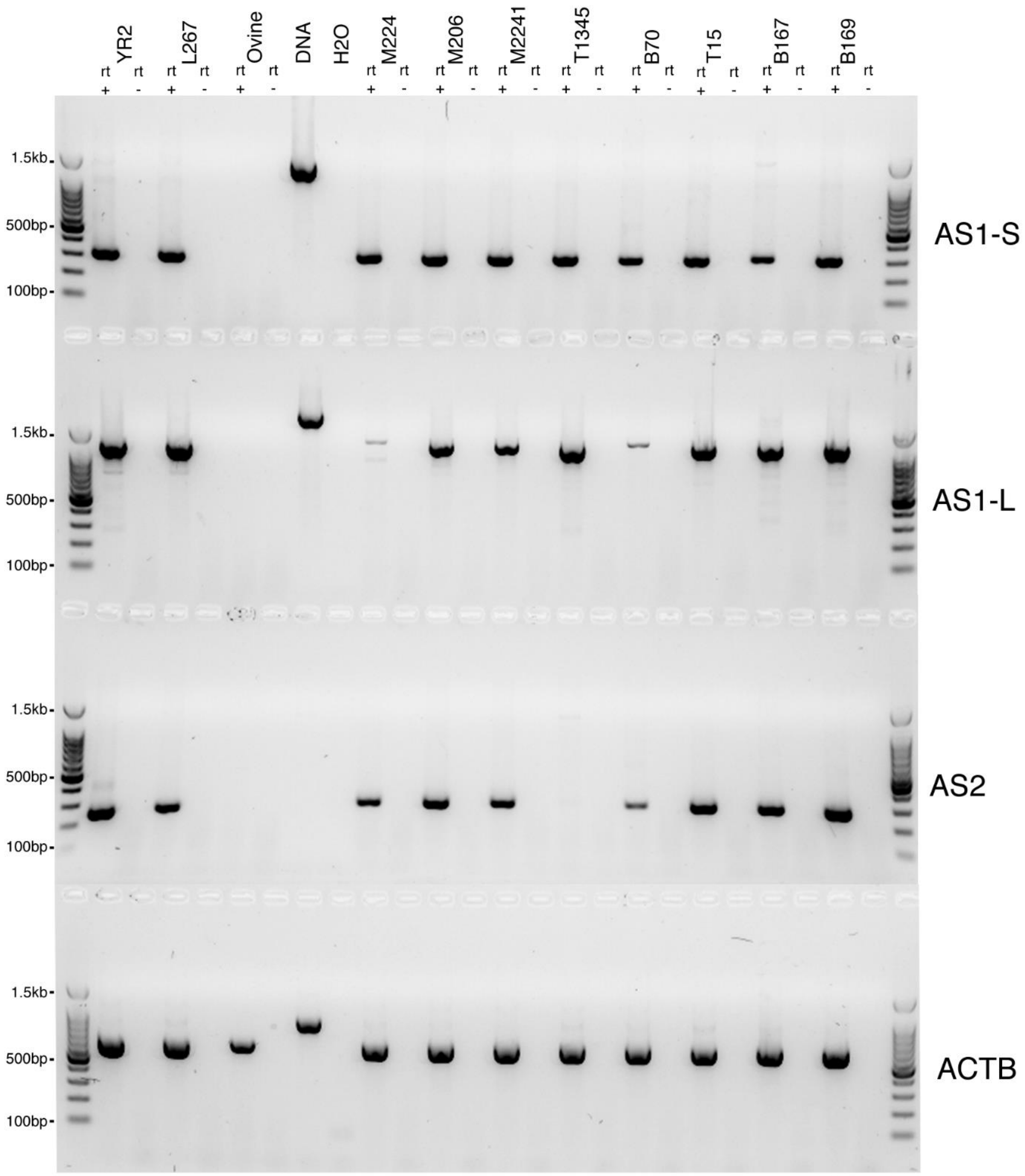

Figure S1 RT-PCR in bovine and ovine tumors. Extended version of Figure 1 B showing RT-PCR results for three ovine (M224, M206, M2241) and five bovine (T1345, B70, T15, B167, B169) tumors. The lack of amplification in T1345 for AS2 is expected as this tumor carries a provirus with a large internal deletion that removes the second exon of AS2 (See Figure S5). (Rt+ reverse transcriptase positive, Rt- reverse transcriptase negative).

Figure S2

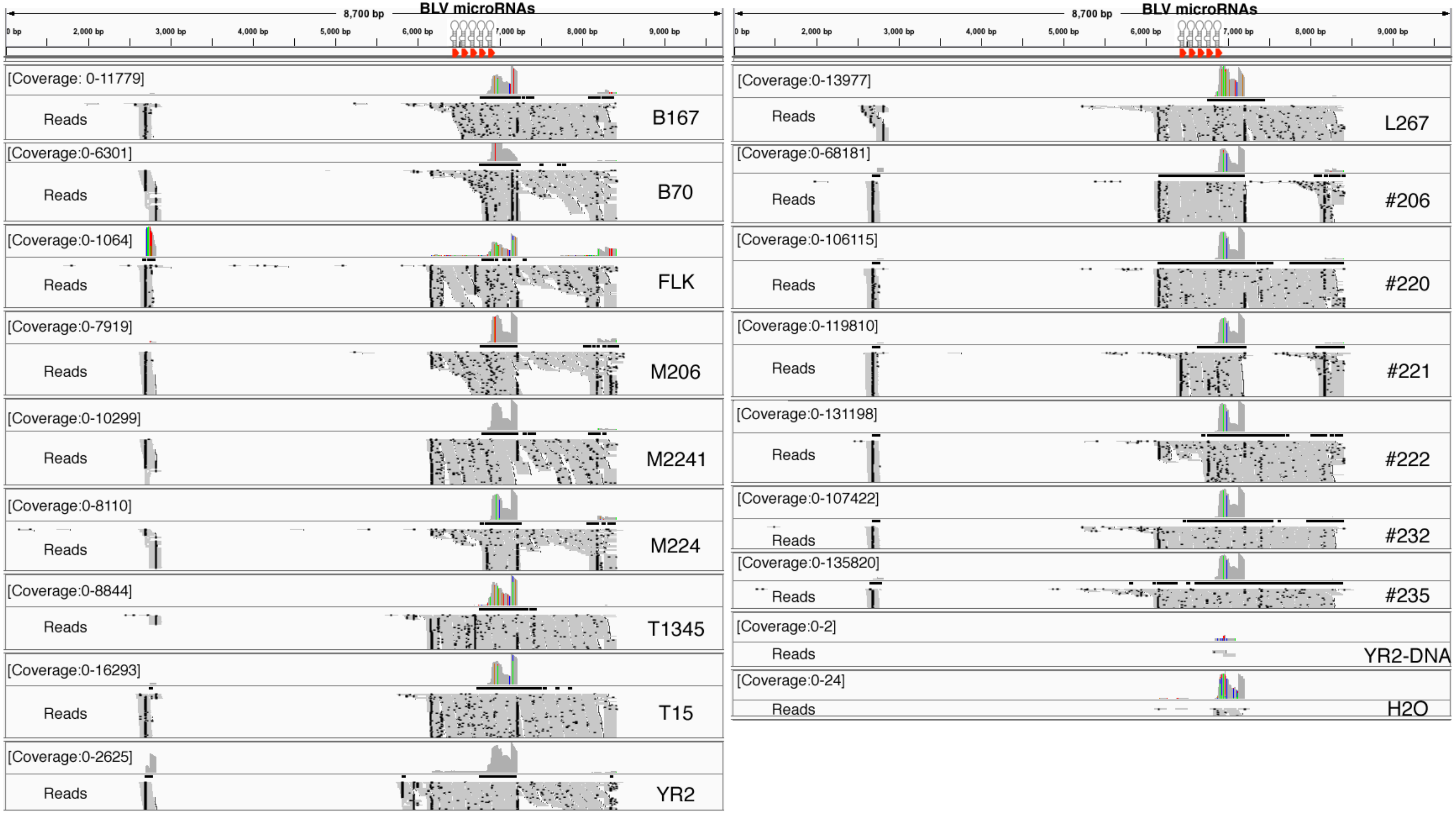

8,700 bp
BLV microRNAs
0 bp
2,000 bp
3,000 bp
4,000 bp
5,000 bp
6,000 bp
7,000 bp
8,000 bp
9,000 bp
[Coverage: 0-11779]
Reads
B167
[Coverage:0-6301]
Reads
B70
[Coverage:0-1064]
Reads
FLK
[Coverage:0-7919]
Reads
M206
[Coverage:0-10299]
Reads
M2241
[Coverage:0-8110]
Reads
M224
[Coverage:0-8844]
Reads
T1345
[Coverage:0-16293]
Reads
T15
[Coverage:0-2625]
Reads
YR2
[Coverage:0-13977]
Reads
L267
[Coverage:0-68181]
Reads
#206
[Coverage:0-106115]
Reads
#220
[Coverage:0-119810]
Reads
#221
[Coverage:0-131198]
Reads
#222
[Coverage:0-107422]
Reads
#232
[Coverage:0-135820]
Reads
#235
[Coverage:0-2]
Reads
YR2-DNA
[Coverage:0-24]
Reads
H2O

Figure S2 Eighteen 3'RACE libraries mapped to the BLV genome. Screen shot from the Integrative Genomics Viewer (IGV) with the position of the BLV microRNAs indicated, showing reads mapping to the BLV genome for cell lines YR2, L267, FLK, three ovine tumors, four bovine tumors and PBMCs isolated at 17 months post-inoculation from 6 asymptomatic BLV infected sheep. In each library, coverage is concentrated immediately following the BLV microRNA region marking the 3' end of AS1-S. A smaller fraction of reads extend beyond the BLV microRNA region (AS1-L) or are spliced further upstream (AS2). As a negative control, libraries were prepared using YR2 DNA and H2O as template. In both of these libraries a small number of reads were observed, due to either cross contamination between samples during the library preparation or inaccurate demultiplexing. In the case of both negative control libraries, the coverage was orders or magnitude lower than that seen in the BLV positive libraries.

Figure S3

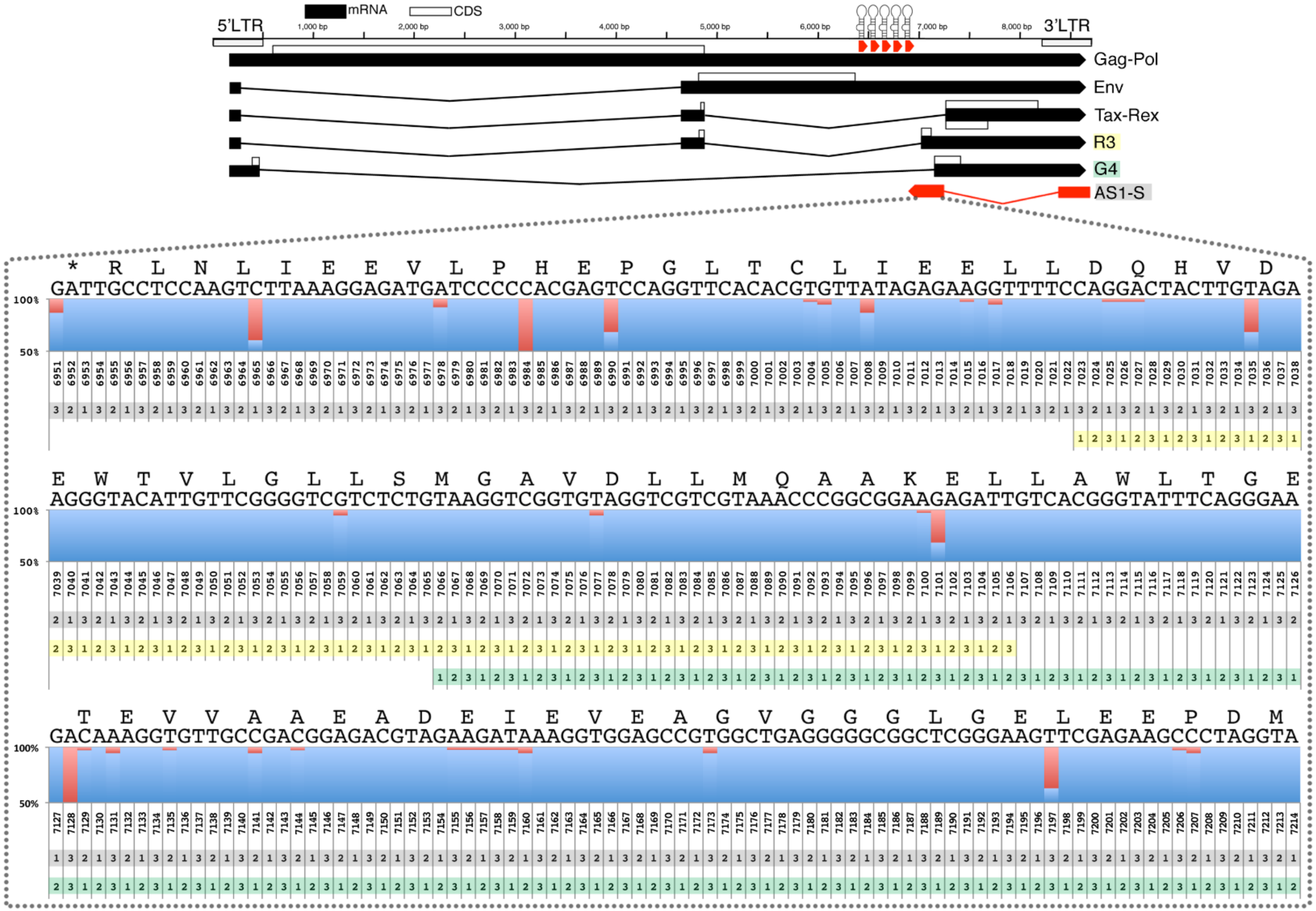

mRNA
CDS
5'LTR
3'LTR
1,000 bp
2,000 bp
3,000 bp
4,000 bp
5,000 bp
6,000 bp
7,000 bp
8,000 bp
Gag-Pol
Env
Tax-Rex
R3
G4
AS1-S
* R L N L I E E V L P H E P G L T C L I E E L L D Q H V D
GATTGCCTCCAAGTCTTAAAGGAGATGATCCCCCACGAGTCCAGGTTCACACGTGTTATAGAGAAGGTTTTCCAGGACTACTTGTAGA
E W T V L G L L S M G A V D L L M Q A A K E L L A W L T G E
AGGGTACATTGTTCGGGGTCGTCTCTGTAAGGTCGGTGTAGGTCGTCGTAAACCCGGCGGAAGAGATTGTCACGGGTATTTCAGGGAA
T E V V A A E A D E I E V E A G V G G G L G E L E E P D M
GACAAAGGTGTTGCCGACGGAGACGTAGAAGATAAAGGTGGAGCCGTGGCTGAGGGGGCGGCTCGGGAAGTTCGAGAAGCCCTAGGTA
100%
50%

Figure S3 BLV AS1 potential open reading frame. Potential amino acid sequence and the underlying nucleotide sequence. Shown below is the percentage conservation for each base calculated using the consensus sequence of the ovine and bovine tumors sequenced by us, as well as six BLV genomes available in NCBI. The amount of red at each position indicates the degree that a particular base varies in the samples examined. Below each base is indicated whether it corresponds to the 1st, 2nd or 3rd position of the relevant ORF. Shown ORFs include the potential AS1 ORF (grey), the sense R3 (yellow) and G4 (green) ORFs.

Figure S4

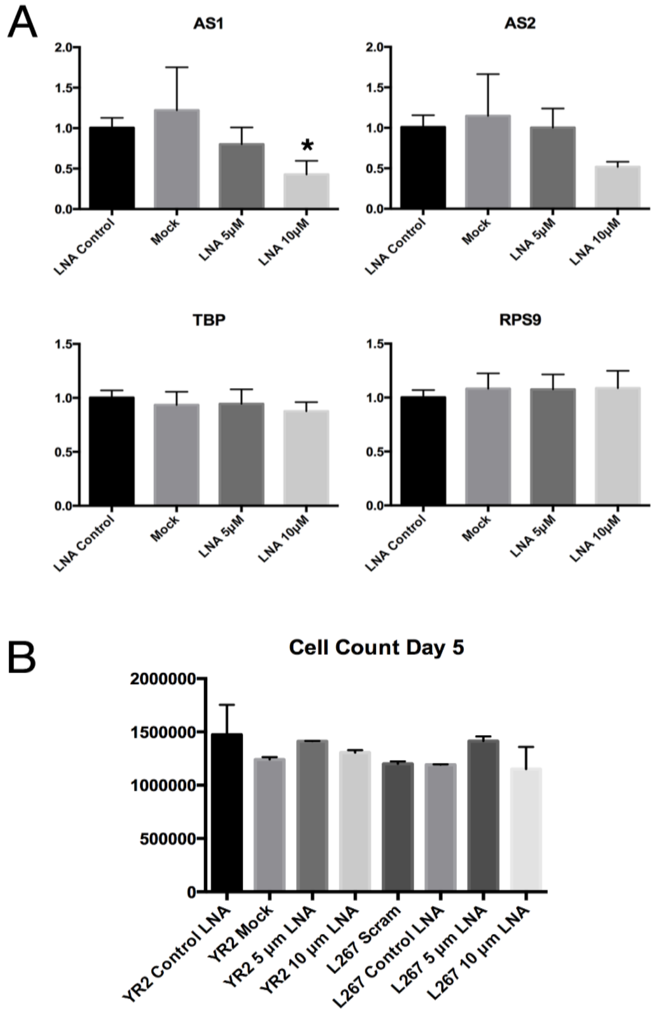

Figure S4 Knock down of AS1 with locked nucleic acids. (A) Three locked nucleic acids (LNAs) designed to target the common first exon of AS1/2, the AS1 splice junction and the second exon of AS1 were mixed and introduced via unassisted uptake at 5 µM and 10 µM concentrations, in duplicate to the YR2 and L267 BLV cell lines. Scrambled LNAs at a concentration of 10 µM and a mock treatment were also carried out. Following 5 days of incubation, RNA was extracted and real time PCR carried out with assays for AS1, AS2, Tax and the house keeping genes RSP9 and TBP. Fold changes observed were scaled to the LNA control samples, which were set to 1. The Kruskal-Wallis test showed a significant difference between the treatments in AS1 (p-value = 0.0192), with Dunn's multiple comparisons test showing a significant difference in LNA Control vs. LNA 10µM (adjusted *p-value < 0.05). For AS2 the Kruskal-Wallis test showed a significant difference between the groups, but Dunn's multiple comparisons test did not show any significance between the individual treatments. Tax expression was not detected in the treated or control YR2 and L267 cells and was not graphed.

(Values are means ± SD combined for YR2 and L267). (B) Following five days of incubation there was no obvious difference in the cell count among the different treatment conditions for both cell lines (data presented are average values and error bar shows the maximum cell count).

Figure S5

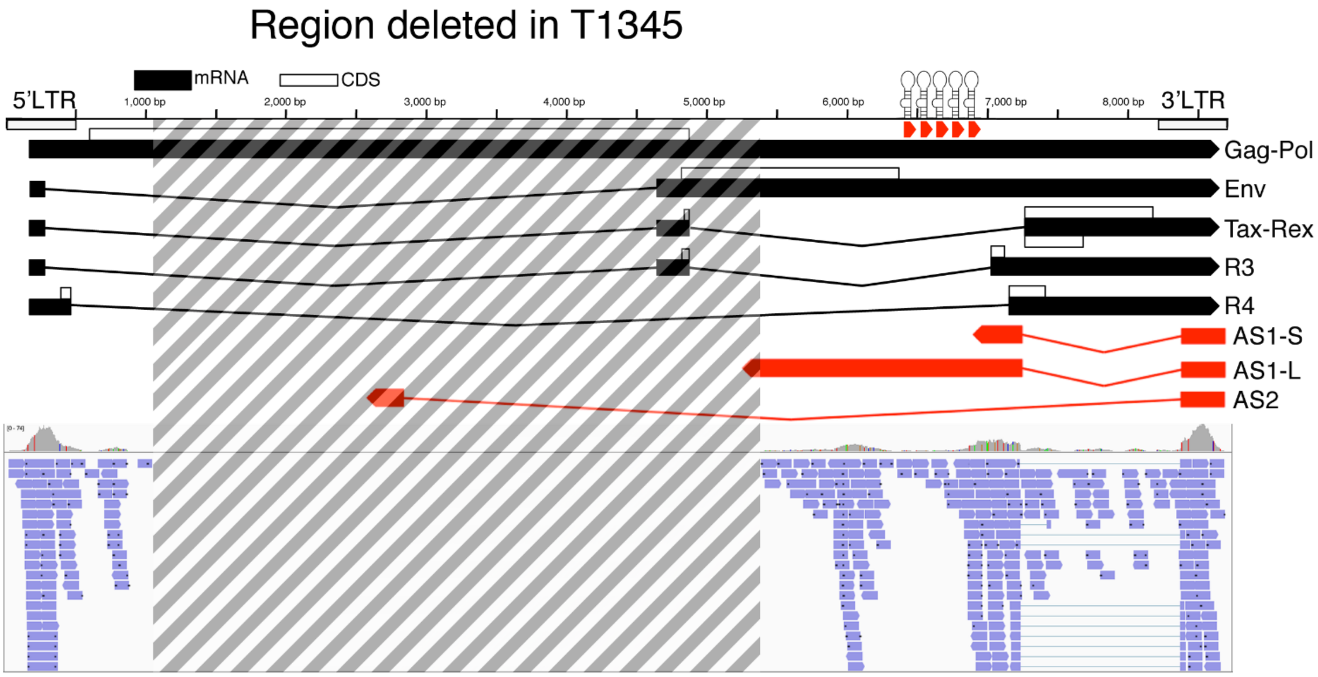

Figure S5 Continued antisense transcription in the defective provirus T1345. Ideogram showing the 4.4kb region deleted (dashed lines) in the T1345 provirus, identified in a monoclonal bovine tumor originating from a natural infection. Despite the deletion and the removal of the potential PAS for AS1-L, antisense transcription remains robust.

Table S1 RNA sequencing results (CPM = counts per million reads)

BLV cell lines	Antisense reads	Sense reads	Uniquely mapped reads	Antisense CPM	Sense CPM	AS CPM / S CPM
L267	732	49	98989055	7.4	0.5	14.9
YR2	526	9	54121243	9.7	0.2	58.4

BLV cell lines Constitutive Tax expressing	Antisense reads	Sense reads*	Uniquely mapped reads	Antisense CPM	Sense CPM*	AS CPM / S CPM*
L267_TaxSN	149	40918	40848268	4	1002	0
YR2_TaxSN	766	2126142	26093199	29	81483	0

*As these cells have been transduced with a lentiviral vector containing the BLV Tax gene, the sense read count will be somewhat artificially elevated.

Ovine Tumors	Antisense reads	Sense reads	Uniquely mapped reads	Antisense CPM	Sense CPM	AS CPM / S CPM
M107	149	1	49133967	3	0	149
M108	10176	96	89870131	113	1	106
M126	416	0	54523313	8	0	na
M138	9734	113	78184059	125	1	86
M155	4922	124	47933750	103	3	40
M160	303	1	26665476	11	0	303
M161	27	1	60630935	0	0	27
M206	323	69	72283867	4	1	5
M20PS	134	0	64457064	2	0	na
M210	531	152	98718132	5	2	3
M21PS	261	0	88207690	3	0	na
M2233	10826	1894	98091530	110	19	6
M2241	333	96	116416582	3	1	3
M224	200	52	123827745	2	0	4
M2311RM	51	1	18352207	3	0	51
M251	476	2	57534668	8	0	238
M2531T	77	23	22379496	3	1	3
M2532PS	90	43	27152057	3	2	2
M28PS	153	2	27420479	6	0	77
M31Leu	134	49	44855981	3	1	3
M32Leu	154	34	43549560	4	1	5
M33Leu	80	177	27828214	3	6	0
M344t0	113	6	26399373	4	0	19
M360	95	11	31508541	3	0	9
M395t0	118	14	25704049	5	1	8
M41Leu	248	99	59025661	4	2	3
M51	145	26	43602381	3	1	6
M5578	154	1	56526611	3	0	154

Bovine Tumors	Antisense reads	Sense reads	Uniquely mapped reads	Antisense CPM	Sense CPM	AS CPM / S CPM
B167	466	104	58806726	7.9	1.8	4.5
B169	372	13	45113265	8.2	0.3	28.6
B3171	358	7	66728945	5.4	0.1	51.1
B3191	736	14	66654840	11.0	0.2	52.6
B3198	212	104	57163314	3.7	1.8	2.0
B3261	270	315	27374722	9.9	11.5	0.9
B70	27	0	45802524	0.6	0.0	na
B76SUS	84	1	81850287	1.0	0.0	84.0
LB120	94	4	43108562	2.2	0.1	23.5
T1345	171	60	37133243	4.6	1.6	2.9
T15	171	60	37133243	4.6	1.6	2.9
B1351	775	1222	147025250	5.3	8.3	0.6

Bovine negative control	Antisense reads	Sense reads	Uniquely mapped reads	Antisense CPM	Sense CPM	AS CPM / S CPM
B78t0	0	0	64282590	0.0	0.0	na

Asymptomatic ovine samples	Antisense reads	Sense reads	Uniquely mapped reads	Antisense CPM	Sense CPM	AS CPM / S CPM	Proviral Load	Number of clones	Largest clone % of reads
#206	31	5	50636602	0.6	0.1	6.2	5.3	1044	4%
#220	32	14	39135557	0.8	0.4	2.3	6.4	810	5%
#221	76	16	41934579	1.8	0.4	4.8	15.5	2118	1%
#222	34	8	38819095	0.9	0.2	4.3	7.4	1522	1%
#232	64	10	40063428	1.6	0.2	6.4	11.2	1368	4%
#235	354	24	58371371	6.1	0.4	14.8	40.6	446	87%

Table S1 continued

BLV microRNA levels in three asymptomatic ovine samples

	#206		#220		#232		Average CPM
	Reads	CPM	Reads	CPM	Reads	CPM	
**BLV-miR-1-3p**	155	79.31	39998	2859.1	24854	1926.96	**1621.79**
**BLV-miR-1-5p**	68	34.79	902	64.48	691	53.57	**50.95**
**BLV-miR-2-3p**	31	15.86	10598	757.56	3834	297.25	**356.89**
**BLV-miR-2-5p**	4544	2325.08	246282	17604.51	107735	8352.81	**9427.47**
**BLV-miR-3-3p**	267	136.62	79434	5678.03	21722	1684.13	**2499.59**
**BLV-miR-3-5p**	1148	587.41	9333	667.13	3680	285.31	**513.28**
**BLV-miR-4-3p**	20887	10687.49	441945	31590.73	248436	19261.52	**20513.25**
**BLV-miR-4-5p**	595	304.45	9210	658.34	5739	444.95	**469.25**
**BLV-miR-5-3p**	3848	1968.95	51727	3697.5	17401	1349.12	**2338.52**
**BLV-miR-5-5p**	15422	7891.15	220195	15739.79	91538	7097.04	**10242.66**

High throughput screening of 5'RACE

Start position	AS1 %	AS2 %
**8661**	3.33	0.00
**8659**	1.13	0.00
**8657**	1.53	0.00
**8656**	0.51	1.80
**8655**	1.42	0.74
**8653**	0.00	2.21
**8650**	42.26	45.88
**8649**	3.29	4.36
**8645**	1.95	0.00
**8640**	0.00	2.39
**8638**	0.62	0.00
**8626**	0.64	0.00
**8622**	0.01	2.03
**8621**	0.67	0.00
**8614**	0.52	0.00
**8598**	2.23	0.00
**8593**	0.90	5.98
**8591**	27.35	20.76
**8590**	3.68	0.00
**8588**	3.69	3.65
**8586**	2.58	5.98
**8578**	0.88	4.24
**8563**	0.80	0.00

Underlined in the U5 portion of the LTR
Non Underlined in the R portion of the LTR

Percentage of reads in the BLV miRNA region that show evidence of cleave mediated via each miRNA (between the 10 and 11bp of the mature microRNA)

	YR2 Total	YR2 nuc	YR2 cyto	#235
**BLV-miR-1-3p**	0	0	0	0
**BLV-miR-1-5p**	35.71	11.91	26.21	19.68
**BLV-miR-2-3p**	3.38	2.00	3.97	2.33
**BLV-miR-2-5p**	7.55	1.59	8.50	0.56
**BLV-miR-3-3p**	0.22	0.00	0.00	0.00
**BLV-miR-3-5p**	0.00	0.00	0.00	0.00
**BLV-miR-4-3p**	0.00	0.00	0.00	0.00
**BLV-miR-4-5p**	16.16	1.22	4.68	3.09
**BLV-miR-5-3p**	0.23	0.03	0.02	0.00
**BLV-miR-5-5p**	2.90	0.34	10.61	0.01
**Total %**	66.14	17.08	53.99	25.67

Table S2 Output of Coding Potential Assessment Tool (CPAT) for Human and Mouse models.

Sequence Name	RNA size	ORF size	Ficket Score Human	Hexamer Score Human	Coding Probability Human	Coding Label Human	Ficket Score Mouse	Hexamer Score mouse	Coding Probability Mouse	Coding Label Mouse	Full description
BLV_ENV_CDS	1548	1548	0.4913	0.0524733	1.000	yes	0.4913	0.054735	0.999	yes	AY078387.1 Bovine leukemia virus envelope glycoprotein polyprotein precursor gPr72 mRNA, complete cds
BLV_TAX_CDS	1323	930	0.7419	0.07244925	0.991	yes	0.7419	0.061266	0.955	yes	M26772.1 BLVTAP Bovine leukemia virus transactivator protein (XBL-1) mRNA, complete cds
AS1-L_BLV	2279	264	1.2074	0.20939568	0.449	yes	1.2074	0.18289	0.386	no	YR2 Region included: 5237-7217 & 8353-8650
AS1-S_BLV	621	264	1.2074	0.20939568	0.474	yes	1.2074	0.18289	0.393	no	YR2 Region included: 6895-7217 & 8353-8650
AS2_BLV	475	24	0.6636	-0.0202475	0.003	no	0.6636	0.043919	0.022	no	YR2 Region included: 2653-2829 & 8353-8650
HTLV1_TAX_CDS	1062	1062	0.6939	0.09265053	0.998	yes	0.6939	0.0917	0.983	yes	AB038239.1 Human T-cell lymphotropic virus type 1 proviral gene for TAX, complete cds
HBZ-SI	1088	621	0.9541	0.3919547	0.984	yes	0.9541	0.388948	0.919	yes	AB219938.1 Human T-lymphotropic virus 1 HBZ-SI mRNA for bZIP factor splicing isoform, complete cds
HBZ	2271	621	0.9541	0.39313014	0.983	yes	0.9541	0.39132	0.918	yes	DQ273132.1 Human T-lymphotropic virus 1 bZIP factor (HBZ) mRNA, complete cds
URS0000627204	126	75	0.8738	0.05856478	0.015	no	0.8738	0.106084	0.060	no	Bos taurus FMR1 antisense RNA 1 conserved region 2
URS0000629A5A	74	39	0.814	0.27273758	0.035	no	0.814	0.26024	0.071	no	Bos taurus ST7 overlapping transcript 3 conserved region 1
URS000062C20C	267	54	0.702	-0.4533927	0.000	no	0.702	-0.40761	0.006	no	Bos taurus Rhabdomyosarcoma 2 associated transcript conserved region 7
URS000062D38D	173	96	0.8215	-0.1728042	0.004	no	0.8215	-0.20799	0.021	no	Bos taurus SMAD5 antisense RNA 1 conserved region 4
URS000062E2FC	184	42	1.0161	0.39283106	0.134	no	1.0161	0.391101	0.159	no	Bos taurus Pvt1 oncogene conserved region 2
URS00006358D2	122	0	0	0	0.000	no	0	0	0.004	no	Bos taurus WT1 antisense RNA conserved region 6
URS000063C6A6	69	60	1.2062	0.31121489	0.167	no	1.2062	0.294404	0.181	no	Bos taurus HOTAIR conserved region 2
URS000063F560	75	66	1.0881	0.31642329	0.132	no	1.0881	0.320309	0.167	no	Bos taurus Six3os1 conserved region 3
URS0000646539	135	21	0.8415	-0.4974953	0.000	no	0.8415	-0.48419	0.005	no	Bos taurus TTC28 antisense RNA 1 conserved region 2
URS000064CDD2	76	12	0.6466	-1.2066197	0.000	no	0.6466	-1.21027	0.000	no	Bos taurus Ghrelin opposite strand RNA conserved region
URS000064D724	91	0	0	0	0.000	no	0	0	0.004	no	Bos taurus SPRY4-IT1 conserved region 1
URS00006510A8	128	0	0	0	0.000	no	0	0	0.004	no	Bos taurus HOX antisense intergenic RNA myeloid 1 conserved region 1
URS0000654C49	128	108	1.0217	-0.1023593	0.012	no	1.0217	-0.12115	0.045	no	Bos taurus ZEB2 antisense RNA 1 conserved region 1
URS0000656B63	196	66	0.9235	-0.4407478	0.001	no	0.9235	-0.50966	0.007	no	Bos taurus DLG2 antisense RNA 1 conserved region 1
URS000065971F	198	132	1.0106	0.38695776	0.279	no	1.0106	0.413437	0.281	no	Bos taurus FTX transcript, XIST regulator conserved region 5
URS000065978A	129	6	0.3621	0.5274011	0.032	no	0.3621	0.478289	0.052	no	Bos taurus Rhabdomyosarcoma 2 associated transcript conserved region 5
URS00006599B8	410	63	0.463	-0.2440055	0.001	no	0.463	-0.22584	0.008	no	Bos taurus Prostate cancer antigen RNA 3 conserved region 2
URS000067674B	226	54	0.9351	-0.209365	0.003	no	0.9351	-0.24973	0.016	no	Bos taurus KCNQ1 overlapping transcript 1 conserved region 5
URS0000677082	362	192	0.6974	0.06993743	0.033	no	0.6974	0.086216	0.090	no	Bos taurus HOXA transcript at the distal tip, conserved region 3
URS0000677825	110	0	0	0	0.000	no	0	0	0.004	no	Bos taurus WT1 antisense RNA conserved region 1
URS00006791CF	71	24	0.8356	-0.2796512	0.001	no	0.8356	-0.26156	0.011	no	Bos taurus FTX transcript, XIST regulator conserved region 1
URS000067BA49	69	27	0.7868	-0.0096499	0.004	no	0.7868	0.012107	0.026	no	Bos taurus SOX2 overlapping transcript exon 2
URS000067BA92	143	9	0.3728	-1.3586378	0.000	no	0.3728	-1.36827	0.000	no	Bos taurus HOX antisense intergenic RNA myeloid 1 conserved region 5
URS000067C9B9	110	0	0	0	0.000	no	0	0	0.004	no	Bos taurus SOX2 overlapping transcript exon 4
URS000067F6F2	95	36	0.7352	-0.4349202	0.000	no	0.7352	-0.48489	0.004	no	Bos taurus Pvt1 oncogene conserved region 3
URS00006863C9	465	132	0.6392	-0.1553791	0.003	no	0.6392	-0.16905	0.022	no	Bos taurus KCNQ1 overlapping transcript 1 conserved region 1
URS0000692A75	272	75	1.0068	-0.3422251	0.002	no	1.0068	-0.35497	0.015	no	Bos taurus ST7 overlapping transcript 4 conserved region 3

Table S2 continued											
URS00006940C3	350	48	1.0193	0.22573242	0.052	no	1.0193	0.219188	0.095	no	Bos taurus Six3os1 conserved region 4
URS0000696811	244	75	0.8859	-0.1532851	0.004	no	0.8859	-0.1798	0.022	no	Bos taurus MEST intronic transcript 1, antisense RNA conserved region
URS000069B56D	77	0	0	0	0.000	no	0	0	0.004	no	Bos taurus MER1 repeat containing imprinted transcript 1 conserved region 1
URS00006B0AAD	142	126	0.5035	-0.1773767	0.002	no	0.5035	-0.22026	0.013	no	Bos taurus Embryonic ventral forebrain-2 long ncRNA
URS00006B3397	139	0	0	0	0.000	no	0	0	0.004	no	Bos taurus T-cell leukemia/lymphoma 6 conserved region 3
URS00006B4586	96	57	0.8308	-0.0128911	0.007	no	0.8308	-0.00722	0.033	no	Bos taurus FTX transcript, XIST regulator conserved region 2
URS00006B4E0E	85	0	0	0	0.000	no	0	0	0.004	no	Bos taurus X-chromosome inactivation gene exon 1
URS00006B5CAE	87	84	0.9455	-0.0385016	0.011	no	0.9455	-0.00353	0.050	no	Bos taurus Six3os1 conserved region 1
URS00006BA2BF	113	45	1.2098	-0.5166496	0.001	no	1.2098	-0.47865	0.012	no	Bos taurus FAM13A antisense RNA 1 conserved region 2
URS00006BE61D	90	42	0.7436	-0.1707097	0.002	no	0.7436	-0.25714	0.010	no	Bos taurus Deleted in lymphocytic leukemia 2 conserved region 2
URS00006C4A69	55	24	0.9172	-0.5042849	0.000	no	0.9172	-0.53981	0.004	no	Bos taurus Deleted in lymphocytic leukemia 2 conserved region 4
URS00006C6BE4	97	15	0.3752	-0.5360566	0.000	no	0.3752	-0.64961	0.001	no	Bos taurus Maternally expressed 8 exon 1
URS00006CA376	106	48	1.2192	0.05717137	0.033	no	1.2192	0.020792	0.071	no	Bos taurus Rhabdomyosarcoma 2 associated transcript conserved region 4
URS00006CB17F	70	6	0.4615	0.5002443	0.036	no	0.4615	0.45189	0.057	no	Bos taurus H19 conserved region 1
URS00006CC536	168	9	0.876	-1.8762211	0.000	no	0.876	-1.53144	0.000	no	Bos taurus FAS antisense RNA 1 conserved region
URS00006CDA32	281	30	0.4802	-0.1819694	0.001	no	0.4802	-0.22201	0.006	no	Bos taurus Highly up-regulated in liver cancer conserved region
URS00006CF3EB	105	9	0.6632	-0.9039742	0.000	no	0.6632	-0.98346	0.000	no	Bos taurus Maternally expressed 3 conserved region 2
URS00006D1977	127	0	0	0	0.000	no	0	0	0.004	no	Bos taurus Nuclear enriched abundant transcript 1 conserved region 2
URS00006D2BA2	108	0	0	0	0.000	no	0	0	0.004	no	Bos taurus Maternally expressed 8 exon 2
URS00006D5400	231	69	0.9188	-0.4056706	0.001	no	0.9188	-0.43784	0.009	no	Bos taurus HOXA11 antisense RNA 1 conserved region 4
URS00006D9004	237	9	0.6218	-1.8230951	0.000	no	0.6218	-1.55709	0.000	no	Bos taurus Hydatidiform mole associated and imprinted conserved region
URS00006DB21C	229	57	0.9927	-0.1059787	0.006	no	0.9927	-0.10886	0.031	no	Bos taurus SOX2 overlapping transcript exon 1
URS00006DD07E	129	114	1.0023	-0.0866744	0.013	no	1.0023	-0.08125	0.052	no	Bos taurus SMAD5 antisense RNA 1 conserved region 2
URS00006DEB8C	288	57	1.1384	-0.2507425	0.004	no	1.1384	-0.14768	0.036	no	Bos taurus WT1 antisense RNA conserved region 7
URS00006E1889	103	39	0.9909	0.21341658	0.041	no	0.9909	0.264031	0.099	no	Bos taurus T-cell leukemia/lymphoma 6 conserved region 1
URS00006E497F	91	87	0.6421	0.08119545	0.010	no	0.6421	0.064335	0.037	no	Bos taurus ZNFX1 antisense RNA 1 conserved region 2
URS00006E59D7	144	72	0.9981	-0.1669965	0.005	no	0.9981	-0.14338	0.031	no	Bos taurus Taurine upregulated gene 1 conserved region 1
URS00006EA6C7	180	0	0	0	0.000	no	0	0	0.004	no	Bos taurus Embryonic ventral forebrain RNA 1 conserved region 1
URS00006EA80E	115	0	0	0	0.000	no	0	0	0.004	no	Bos taurus FAM13A antisense RNA 1 conserved region 1
URS00006EEA29	135	42	1.0223	-0.1295657	0.005	no	1.0223	-0.09857	0.031	no	Bos taurus Myocardial infarction associated transcript exon 1
URS00006F4484	68	0	0	0	0.000	no	0	0	0.004	no	Bos taurus Maternally expressed 8 exon 3
URS00006F60BE	92	78	1.1896	-0.0083285	0.027	no	1.1896	0.019669	0.082	no	Bos taurus ZNFX1 antisense RNA 1 conserved region 2
URS00006F7F53	154	48	1.1266	-0.0075192	0.016	no	1.1266	0.045069	0.065	no	Bos taurus NPPA antisense RNA 1 conserved region 2
URS00006FE4FF	171	75	0.779	-0.3315217	0.001	no	0.779	-0.36448	0.009	no	Bos taurus ST7 overlapping transcript 4 conserved region 2
URS00006FF6E6	143	99	0.8323	0.11958267	0.026	no	0.8323	0.150557	0.076	no	Bos taurus Rhabdomyosarcoma 2 associated transcript conserved region 8
URS00006FFF5E	262	114	1.014	0.05805101	0.035	no	1.014	0.042241	0.081	no	Bos taurus SMAD5 antisense RNA 1 conserved region 1
URS00007035F2	132	18	0.408	-0.0844732	0.001	no	0.408	-0.18755	0.006	no	Bos taurus Pvt1 oncogene conserved region 5
URS00007036C6	141	57	0.6862	0.02145747	0.006	no	0.6862	0.067528	0.033	no	Bos taurus Rhabdomyosarcoma 2 associated transcript conserved region 2
URS0000708ABD	586	120	0.8006	0.07255146	0.021	no	0.8006	0.115562	0.073	no	Bos taurus Vax2os1 conserved region 2

Table S2 continued											
URS0000717AE7	102	0	0	0	0.000	no	0	0	0.004	no	Bos taurus ZEB2 antisense RNA 1 conserved region 3
URS0000717FE0	101	0	0	0	0.000	no	0	0	0.004	no	Bos taurus ZEB2 antisense RNA 1 conserved region 3
URS0000719B0F	77	48	1.1266	-0.0075192	0.016	no	1.1266	0.045069	0.065	no	Bos taurus NPPA antisense RNA 1 conserved region 2
URS00007259BA	104	75	1.0494	-0.2156819	0.004	no	1.0494	-0.21204	0.027	no	Bos taurus Metastasis associated lung adenocarcinoma transcript 1
URS0000726CC0	118	12	1.0582	-0.380929	0.001	no	1.0582	-0.48892	0.007	no	Bos taurus GNAS antisense RNA 1 conserved region 2
URS000072850B	247	45	1.1273	-0.2826858	0.003	no	1.1273	-0.28376	0.020	no	Bos taurus Prostate androgen-regulated transcript 1 conserved region 2
URS000072AE6B	54	27	0.867	-0.7723672	0.000	no	0.867	-0.8099	0.002	no	Bos taurus FMR1 antisense RNA 1 conserved region 1
URS000072B940	215	192	1.0975	0.05917406	0.100	no	1.0975	0.060013	0.165	no	Bos taurus DLG2 antisense RNA 1 conserved region 2
URS000072CEB9	330	141	0.9852	-0.0307435	0.024	no	0.9852	-0.01007	0.078	no	Bos taurus Deleted in lymphocytic leukemia 1 conserved region 2
URS0000759C73	2069	345	0.5704	0.12308877	0.132	no	0.5704	0.166592	0.234	no	Bos taurus H19, imprinted maternally expressed transcript (non-protein coding) (H19), long non-coding RNA.
URS0000759E04	539	348	1.0681	0.42958035	0.861	yes	1.0681	0.408492	0.675	yes	Bos taurus artiodactyl-specific transcript 1 (AST1), long non-coding RNA.
URS000075A1EC	978	177	0.9069	0.16822608	0.093	no	0.9069	0.171259	0.153	no	Bos taurus uncharacterized LOC100196901 (LOC100196901), transcript variant 1, long non-coding RNA.
URS000075A42C	801	282	0.6385	-0.0187509	0.039	no	0.6385	0.026889	0.119	no	Bos taurus uncharacterized LOC100847825 (LOC100847825), long non-coding RNA.
URS000075B4CA	1135	180	0.6608	-0.0817498	0.009	no	0.6608	-0.08575	0.042	no	Bos taurus chromosome 23 open reading frame, human C6orf52 (C23H6orf52), transcript variant 1, long non-coding RNA.
URS000075B604	1008	228	0.3596	-0.2278236	0.002	no	0.3596	-0.26194	0.018	no	Bos taurus uncharacterized LOC100847946 (LOC100847946), long non-coding RNA.
URS000075B9FD	955	144	0.6445	-0.1496252	0.004	no	0.6445	-0.17596	0.023	no	Bos taurus uncharacterized LOC100848574 (LOC100848574), transcript variant 6, long non-coding RNA.
URS000075BC30	1638	381	0.7	0.06400548	0.189	no	0.7	0.097322	0.286	no	Bos taurus uncharacterized LOC100140121 (LOC100140121), long non-coding RNA.
URS000075C0CE	1954	555	0.7853	0.19222819	0.821	yes	0.7853	0.22794	0.731	yes	Bos taurus maternally expressed 3 (non-protein coding) (MEG3), long non-coding RNA.
URS000075C544	806	180	0.6608	-0.0817498	0.009	no	0.6608	-0.08575	0.042	no	Bos taurus chromosome 23 open reading frame, human C6orf52 (C23H6orf52), transcript variant 3, long non-coding RNA.
URS000075CDC9	446	78	0.8326	-0.1762391	0.003	no	0.8326	-0.21904	0.018	no	Bos taurus small nucleolar RNA host gene 4 (non-protein coding) (SNHG4), long non-coding RNA.
URS000075CF6E	888	165	0.6564	-0.1177284	0.006	no	0.6564	-0.14663	0.030	no	Bos taurus uncharacterized LOC788201 (LOC788201), long non-coding RNA.
URS000075D01A	1078	372	0.5896	0.07532513	0.143	no	0.5896	0.099628	0.235	no	Bos taurus small nucleolar RNA host gene 12 (non-protein coding) (SNHG12), transcript variant 2, long non-coding RNA.
URS000075D08D	546	144	0.8749	0.07291865	0.034	no	0.8749	0.058342	0.081	no	Bos taurus uncharacterized LOC100616526 (LOC100616526), transcript variant 3, long non-coding RNA.
URS000075D922	911	144	0.6445	-0.1496252	0.004	no	0.6445	-0.17596	0.023	no	Bos taurus uncharacterized LOC100848574 (LOC100848574), transcript variant 4, long non-coding RNA.
URS000075DA73	3345	186	0.6977	0.2334901	0.072	no	0.6977	0.296428	0.162	no	Bos taurus uncharacterized LOC782788 (MGC148318), long non-coding RNA.
URS000075DAE4	413	168	0.9531	0.16216371	0.096	no	0.9531	0.159035	0.151	no	Bos taurus uncharacterized LOC100616098 (LOC100616098), long non-coding RNA.

**Table S2 continued**										
URS000075DCA1	3462	342	0.7099	0.08846014	0.142	no	0.7099	0.070938	0.213	no	Bos taurus uncharacterized LOC100616526 (LOC100616526), transcript variant 1, long non-coding RNA.
URS000075E3D6	3025	1380	0.9853	0.32875034	1.000	yes	0.9853	0.32318	1.000	yes	Bos taurus PPARG-TSEN2 (PPARG-TSEN2), transcript variant 1, long non-coding RNA.
URS000075EAE1	1030	180	0.6608	-0.0817498	0.009	no	0.6608	-0.08575	0.042	no	Bos taurus chromosome 23 open reading frame, human C6orf52 (C23H6orf52), transcript variant 2, long non-coding RNA.
URS000075EB28	1123	372	0.5896	0.07532513	0.143	no	0.5896	0.099628	0.235	no	Bos taurus small nucleolar RNA host gene 12 (non-protein coding) (SNHG12), transcript variant 1, long non-coding RNA.
URS000075EC71	1875	1107	0.9629	0.40041855	1.000	yes	0.9629	0.410277	0.998	yes	Bos taurus LOC100271850-MEF2B readthrough transcript (LOC100271850-MEF2B), long non-coding RNA.
URS000075EDB2	1014	147	0.894	0.0876376	0.040	no	0.894	0.048664	0.082	no	Bos taurus uncharacterized LOC100848574 (LOC100848574), transcript variant 2, long non-coding RNA.
URS0000623174	121	72	1.0222	0.23927818	0.073	no	1.0222	0.241506	0.121	no	Bos taurus Nuclear enriched abundant transcript 1 conserved region 1
URS0000625647	155	42	0.6501	-0.1411949	0.001	no	0.6501	-0.13515	0.013	no	Bos taurus Pregnancy induced noncoding RNA
URS0000627F0B	157	45	0.8968	-0.0799771	0.005	no	0.8968	-0.12273	0.023	no	Bos taurus TP53 target 1 conserved region 1
URS0000627F39	113	6	0.3299	-2.901853	0.000	no	0.3299	-3.08032	0.000	no	Bos taurus Pvt1 oncogene conserved region 6
URS000062B719	131	24	0.7015	-0.1406256	0.001	no	0.7015	-0.11695	0.014	no	Bos taurus Prostate-specific transcript 1
URS000062B8A2	204	24	0.4446	0.45463191	0.031	no	0.4446	0.468966	0.067	no	Bos taurus Maternal intergenic circadian oscillating RNA 1
URS000062CBF8	135	15	1.0095	0.08828311	0.015	no	1.0095	0.066005	0.044	no	Bos taurus Deleted in lymphocytic leukemia 2 conserved region 6
URS000062EA6D	102	12	0.3194	-1.5470274	0.000	no	0.3194	-1.53147	0.000	no	Bos taurus HOX antisense intergenic RNA myeloid 1 conserved region 4
URS000062F99B	124	51	0.7689	0.02880937	0.007	no	0.7689	0.007589	0.029	no	Bos taurus Pvt1 oncogene conserved region 3
URS000063B8D5	85	0	0	0	0.000	no	0	0	0.004	no	Bos taurus X-chromosome inactivation gene exon 1
URS000063C398	76	0	0	0	0.000	no	0	0	0.004	no	Bos taurus ZNRD1 antisense RNA 1 conserved region 2
URS000063DD01	129	96	0.8591	-0.2202319	0.003	no	0.8591	-0.2425	0.020	no	Bos taurus CDKN2B antisense RNA 1 convserved region 3
URS000063E095	80	75	0.9386	-0.23211	0.003	no	0.9386	-0.23885	0.020	no	Bos taurus Deleted in lymphocytic leukemia 2 conserved region 5
URS000063F98F	146	21	0.7892	-0.4194346	0.000	no	0.7892	-0.41721	0.005	no	Bos taurus Myocardial infarction associated transcript exon 5 conserved region 1
URS0000641959	179	9	0.6695	-1.1386872	0.000	no	0.6695	-1.15941	0.000	no	Bos taurus Pvt1 oncogene conserved region 4
URS0000643C57	50	0	0	0	0.000	no	0	0	0.004	no	Bos taurus H19 conserved region 1
URS000064A264	176	105	0.9142	-0.244312	0.003	no	0.9142	-0.23639	0.024	no	Bos taurus NPPA antisense RNA 1 conserved region 1
URS000064D7DB	115	39	0.7422	-0.4039124	0.000	no	0.7422	-0.36695	0.007	no	Bos taurus KCNQ1 overlapping transcript 1 conserved region 3
URS0000660D10	85	0	0	0	0.000	no	0	0	0.004	no	Bos taurus Myocardial infarction associated transcript exon 5 conserved region 2
URS0000662396	89	60	1.1772	-0.651044	0.000	no	1.1772	-0.70488	0.005	no	Bos taurus Taurine upregulated gene 1 conserved region 2
URS000066403E	107	69	0.5719	-0.3791522	0.000	no	0.5719	-0.35714	0.006	no	Bos taurus Polled intersex syndrome regulated transcript 1
URS00006643D0	215	186	0.5145	0.08017167	0.019	no	0.5145	0.097536	0.064	no	Bos taurus Six3os1 conserved region 2
URS0000665133	237	9	0.6218	-1.8230951	0.000	no	0.6218	-1.55709	0.000	no	Bos taurus Hydatidiform mole associated and imprinted conserved region
URS0000666A1B	92	78	0.9769	0.01201435	0.016	no	0.9769	0.037318	0.059	no	Bos taurus ZNFX1 antisense RNA 1 conserved region 2
URS00006678DE	168	9	0.876	-1.8762211	0.000	no	0.876	-1.53144	0.000	no	Bos taurus FAS antisense RNA 1 conserved region
URS0000668B27	210	39	0.8128	-0.3463585	0.001	no	0.8128	-0.4175	0.006	no	Bos taurus HOXA11 antisense RNA 1 conserved region 5
URS0000669E60	118	57	0.8845	-0.3215334	0.001	no	0.8845	-0.38115	0.010	no	Bos taurus Highly accelerated region 1A/1B
URS0000669F75	150	36	0.7222	-0.1034753	0.002	no	0.7222	-0.11845	0.015	no	Bos taurus Rhabdomyosarcoma 2 associated transcript conserved region 10

Table S2 continued											
URS000066A9DA	206	81	0.8972	-0.3519839	0.001	no	0.8972	-0.39404	0.011	no	Bos taurus ST7 overlapping transcript 4 conserved region 4
URS000066CA78	70	24	0.8507	-0.5007781	0.000	no	0.8507	-0.37277	0.007	no	Bos taurus JPX transcript, XIST activator conserved region 2
URS000066DB40	239	66	1.2803	0.22602503	0.132	no	1.2803	0.221907	0.169	no	Bos taurus Taurine upregulated gene 1 conserved region 3
URS000066F704	277	174	0.7893	0.03494329	0.029	no	0.7893	0.004977	0.071	no	Bos taurus SOX2 overlapping transcript exon 3
URS000066F725	208	63	1.2486	-0.5533622	0.001	no	1.2486	-0.56091	0.011	no	Bos taurus Six3os1 conserved region 7
URS00006703D9	127	12	0.9238	-0.5402292	0.000	no	0.9238	-0.50023	0.005	no	Bos taurus T-cell leukemia/lymphoma 6 conserved region 2
URS0000671CC7	151	60	1.0555	-0.0020796	0.015	no	1.0555	0.017072	0.056	no	Bos taurus FTX transcript, XIST regulator conserved region 3
URS0000673933	177	6	0.3194	-3.3444442	0.000	no	0.3194	-3.14823	0.000	no	Bos taurus Taurine upregulated gene 1 conserved region 4
URS000067E11A	215	72	1.104	0.12378344	0.045	no	1.104	0.144991	0.102	no	Bos taurus DLG2 antisense RNA 1 conserved region 1
URS0000680E2C	185	177	1.0005	0.12838402	0.099	no	1.0005	0.174034	0.182	no	Bos taurus Pvt1 oncogene conserved region 1
URS0000682766	223	6	0.3194	-3.3444442	0.000	no	0.3194	-3.14823	0.000	no	Bos taurus Prostate androgen-regulated transcript 1 conserved region 1
URS00006867A3	69	24	0.8507	-0.5007781	0.000	no	0.8507	-0.37277	0.007	no	Bos taurus JPX transcript, XIST activator conserved region 2
URS000068C47E	123	21	0.6706	-0.9344654	0.000	no	0.6706	-0.9314	0.001	no	Bos taurus SPRY4-IT1 conserved region 2
URS0000691107	97	15	0.3752	-0.5360566	0.000	no	0.3752	-0.64961	0.001	no	Bos taurus Maternally expressed 8 exon 1
URS0000693A09	374	69	0.8801	0.12245743	0.022	no	0.8801	0.177823	0.074	no	Bos taurus HOXA transcript at the distal tip, conserved region 2
URS0000696C1D	135	90	0.7435	-0.0272282	0.007	no	0.7435	-0.01036	0.035	no	Bos taurus HOXB13 antisense RNA 1 conserved region 1
URS0000697ADE	122	0	0	0	0.000	no	0	0	0.004	no	Bos taurus WT1 antisense RNA conserved region 4
URS000069BEA8	98	0	0	0	0.000	no	0	0	0.004	no	Bos taurus Maternally expressed 8 exon 2
URS00006A3960	201	189	0.8598	-0.018075	0.030	no	0.8598	-0.01015	0.085	no	Bos taurus DAOA antisense RNA 1 conserved region 2
URS00006A711E	271	57	0.627	-0.289817	0.001	no	0.627	-0.2342	0.010	no	Bos taurus Six3os1 conserved region 5
URS00006A7D6B	198	0	0	0	0.000	no	0	0	0.004	no	Bos taurus ST7 overlapping transcript 4 conserved region 1
URS00006A961C	61	39	1.0075	0.13282328	0.026	no	1.0075	0.116644	0.062	no	Bos taurus Rhabdomyosarcoma 2 associated transcript conserved region 1
URS00006AAE04	85	36	0.8983	0.35988544	0.075	no	0.8983	0.35483	0.112	no	Bos taurus NPPA antisense RNA 1 conserved region 3
URS00006AD16C	129	87	0.4435	-0.0253377	0.003	no	0.4435	-0.00984	0.019	no	Bos taurus WT1 antisense RNA conserved region 2
URS00006AD459	105	9	0.6632	-0.9039742	0.000	no	0.6632	-0.98346	0.000	no	Bos taurus Maternally expressed 3 conserved region 2
URS00006AE16B	272	21	0.7803	-1.0359179	0.000	no	0.7803	-0.85649	0.001	no	Bos taurus WT1 antisense RNA conserved region 8
URS00006AEBC0	98	0	0	0	0.000	no	0	0	0.004	no	Bos taurus HOTAIR conserved region 3
URS00006B25E5	109	18	0.4709	-0.3128058	0.000	no	0.4709	-0.34822	0.004	no	Bos taurus Rhabdomyosarcoma 2 associated transcript conserved region 3
URS00006B2E3C	166	84	0.7209	0.15808265	0.020	no	0.7209	0.129369	0.052	no	Bos taurus HOXA transcript at the distal tip, conserved region 4
URS00006B6641	202	84	0.8651	-0.0218437	0.010	no	0.8651	-0.04986	0.036	no	Bos taurus Rhabdomyosarcoma 2 associated transcript conserved region 6
URS00006BB24D	217	138	0.9914	0.02906229	0.035	no	0.9914	0.011574	0.083	no	Bos taurus HOX antisense intergenic RNA myeloid 1 conserved region 2
URS00006BE6E9	113	54	0.5573	-0.5017926	0.000	no	0.5573	-0.52344	0.003	no	Bos taurus ST7 overlapping transcript 3 conserved region 3
URS00006C52CB	173	0	0	0	0.000	no	0	0	0.004	no	Bos taurus TTC28 antisense RNA 1 conserved region 3
URS00006C91FB	196	33	0.6767	0.20344683	0.014	no	0.6767	0.271954	0.055	no	Bos taurus Vax2os1 conserved region 3
URS00006CB091	133	30	0.8607	-0.0340372	0.005	no	0.8607	-0.05183	0.025	no	Bos taurus JPX transcript, XIST activator conserved region 1
URS00006CCFE8	99	69	1.0945	0.11334694	0.040	no	1.0945	0.102862	0.086	no	Bos taurus MEST intronic transcript 1, antisense RNA conserved region
URS00006CF2CE	193	90	1.101	0.3749089	0.231	no	1.101	0.311005	0.191	no	Bos taurus Rhabdomyosarcoma 2 associated transcript conserved region 9
URS00006D0C82	383	18	0.9971	-0.6064877	0.000	no	0.9971	-0.46047	0.007	no	Bos taurus Myocardial infarction associated transcript exon 5 conserved region 3
URS00006D2A59	83	36	0.9707	-0.129272	0.004	no	0.9707	-0.1004	0.027	no	Bos taurus NPPA antisense RNA 1 conserved region 3
URS00006D6D45	159	120	0.7575	0.18214124	0.038	no	0.7575	0.191946	0.088	no	Bos taurus Nuclear enriched abundant transcript 1 conserved region 3

Table S2 continued											
URS00006D8830	56	36	0.9322	0.21146634	0.033	no	0.9322	0.255745	0.085	no	Bos taurus HOXA transcript at the distal tip, conserved region 1
URS00006DCB57	191	54	0.6521	-0.3547823	0.000	no	0.6521	-0.35512	0.006	no	Bos taurus Deleted in lymphocytic leukemia 1 conserved region 1
URS00006E0845	38	0	0	0	0.000	no	0	0	0.004	no	Bos taurus GNAS antisense RNA 1 conserved region 3
URS00006E2E2E	92	78	1.1063	0.0950567	0.041	no	1.1063	0.056084	0.079	no	Bos taurus ZNFX1 antisense RNA 1 conserved region 2
URS00006E4C4E	118	0	0	0	0.000	no	0	0	0.004	no	Bos taurus ST7 antisense RNA 1 conserved region 1
URS00006E63B7	107	0	0	0	0.000	no	0	0	0.004	no	Bos taurus Maternally expressed 8 exon 2
URS00006E88AE	71	51	0.6392	-0.2225712	0.001	no	0.6392	-0.27969	0.008	no	Bos taurus FTX transcript, XIST regulator conserved region 1
URS00006FBAD6	179	126	0.9038	-0.2132939	0.005	no	0.9038	-0.16021	0.036	no	Bos taurus HOXA11 antisense RNA 1 conserved region 6
URS00006FEDB4	112	75	0.3852	-0.1964278	0.001	no	0.3852	-0.2353	0.007	no	Bos taurus Non-protein coding RNA, upstream of F2R/PAR1 conserved region 1
URS0000703281	211	39	0.9198	-0.2777535	0.001	no	0.9198	-0.44458	0.007	no	Bos taurus HOXA11 antisense RNA 1 conserved region 5
URS0000703385	76	6	0.3299	-2.901853	0.000	no	0.3299	-3.08032	0.000	no	Bos taurus CLRN1 antisense RNA 1 conserved region
URS0000705FA5	122	18	0.8956	-0.6394999	0.000	no	0.8956	-0.64138	0.003	no	Bos taurus Deleted in lymphocytic leukemia 2 conserved region 1
URS000071003F	98	63	0.8698	0.48692793	0.186	no	0.8698	0.520013	0.210	no	Bos taurus HOXA11 antisense RNA 1 conserved region 3
URS0000710D82	223	60	0.8505	-0.4381931	0.000	no	0.8505	-0.39521	0.009	no	Bos taurus Prostate androgen-regulated transcript 1 conserved region 1
URS00007164B5	194	54	0.7073	-0.1455383	0.002	no	0.7073	-0.24467	0.011	no	Bos taurus KCNQ1 overlapping transcript 1 conserved region 2
URS0000719068	232	78	0.7878	-0.1269287	0.004	no	0.7878	-0.11622	0.024	no	Bos taurus WT1 antisense RNA conserved region 3
URS0000720259	105	0	0	0	0.000	no	0	0	0.004	no	Bos taurus GNAS antisense RNA 1 conserved region 1
URS000072B58A	126	0	0	0	0.000	no	0	0	0.004	no	Bos taurus GNAS antisense RNA 1 conserved region 4
URS000072C9A4	105	9	0.6632	-0.9039742	0.000	no	0.6632	-0.98346	0.000	no	Bos taurus Maternally expressed 3 conserved region 2
URS000072DC14	201	81	0.8558	-0.0937773	0.006	no	0.8558	-0.11216	0.028	no	Bos taurus DAOA antisense RNA 1 conserved region 1
URS0000751D1A	820	324	0.8015	0.07230413	0.161	no	0.8015	0.11947	0.262	no	Bos taurus (cattle) X5
URS0000759E9B	22812	567	0.48	-0.058008	0.097	no	0.48	-0.06463	0.277	no	Bos taurus X (inactive)-specific transcript (XIST), long non-coding RNA.
URS000075A02E	1894	306	0.6476	-0.0680364	0.035	no	0.6476	-0.03546	0.113	no	Bos taurus uncharacterized LOC100848703 (LOC100848703), long non-coding RNA.
URS000075AAC6	930	177	0.9069	0.16822608	0.093	no	0.9069	0.171259	0.153	no	Bos taurus uncharacterized LOC100196901 (LOC100196901), transcript variant 2, long non-coding RNA.
URS000075AD92	751	144	0.6445	-0.1496252	0.004	no	0.6445	-0.17596	0.023	no	Bos taurus uncharacterized LOC100848574 (LOC100848574), transcript variant 7, long non-coding RNA.
URS000075B437	926	177	0.9069	0.16822608	0.093	no	0.9069	0.171259	0.153	no	Bos taurus uncharacterized LOC100196901 (LOC100196901), transcript variant 3, long non-coding RNA.
URS000075B61B	886	144	0.6445	-0.1496252	0.004	no	0.6445	-0.17596	0.023	no	Bos taurus uncharacterized LOC100848574 (LOC100848574), transcript variant 5, long non-coding RNA.
URS000075B744	2851	2676	1.1013	0.33495888	1.000	yes	1.1013	0.329076	1.000	yes	Bos taurus PPARG-TSEN2 (PPARG-TSEN2), transcript variant 2, long non-coding RNA.
URS000075BB85	892	114	0.6841	0.02240328	0.010	no	0.6841	0.042168	0.044	no	Bos taurus antisense IGF2R RNA noncoding (AIRN), long non-coding RNA.
URS000075C9BA	795	111	0.9282	0.05455613	0.025	no	0.9282	0.07886	0.076	no	Bos taurus uncharacterized LOC100616526 (LOC100616526), transcript variant 2, long non-coding RNA.
URS000075CB6C	1120	147	0.894	0.0876376	0.040	no	0.894	0.048664	0.082	no	Bos taurus uncharacterized LOC100848574 (LOC100848574), transcript variant 1, long non-coding RNA.
URS000075DFAF	777	135	0.7837	-0.1091227	0.007	no	0.7837	-0.11944	0.035	no	Bos taurus small nucleolar RNA host gene 3 (non-protein coding) (SNHG3), long non-coding RNA.
URS00001A8549	918	543	1.1996	0.36515742	0.980	yes	1.1996	0.376433	0.908	yes	Bos taurus (cattle) partial PEG11 antisense RNA
URS000061E61B	644	189	0.7462	-0.1219713	0.010	no	0.7462	-0.14049	0.044	no	Bos taurus (cattle) noncoding RNA

Table S2 continued

URS0000626285	61	6	0.3621	1.18215914	0.712	yes	0.3621	1.040412	0.302	no	Bos taurus ZEB2 antisense RNA 1 conserved region 2
URS000062C8E7	67	15	0.6556	0.07351687	0.005	no	0.6556	0.056913	0.022	no	Bos grunniens H19 conserved region 2
URS000063BDFC	58	3	0.3299	0	0.001	no	0.3299	0	0.009	no	Ovis aries HOX antisense intergenic RNA myeloid 1 conserved region 3
URS00006515F5	126	54	0.9473	0.13648592	0.026	no	0.9473	0.149701	0.069	no	Ovis aries X-chromosome inactivation gene exon 4
URS0000667B4F	76	0	0	0	0.000	no	0	0	0.004	no	Ovis aries Deleted in lymphocytic leukemia 2 conserved region 3
URS000067C63C	125	123	1.1507	0.04518516	0.054	no	1.1507	0.049557	0.113	no	Bos taurus H19 conserved region 3
URS000068C069	46	0	0	0	0.000	no	0	0	0.004	no	Ovis aries CDKN2B antisense RNA 1 convserved region 2
URS000069115D	96	48	0.8041	-0.1356044	0.003	no	0.8041	-0.10815	0.020	no	Bos taurus HOXA11 antisense RNA 1 conserved region 1
URS000069C46C	71	33	1.0113	-0.0135459	0.009	no	1.0113	-0.08887	0.029	no	Bos taurus Ghrelin opposite strand RNA conserved region
URS00006A38EC	125	111	0.9834	0.221769	0.087	no	0.9834	0.212901	0.133	no	Bos taurus SMAD5 antisense RNA 1 conserved region 3
URS00006BA301	186	24	0.7776	-0.4064206	0.000	no	0.7776	-0.2448	0.010	no	Ovis aries HOXA11 antisense RNA 1 conserved region 2
URS00006BB01F	140	27	0.5365	-0.3212849	0.000	no	0.5365	-0.44433	0.003	no	Ovis aries FTX transcript, XIST regulator conserved region 4
URS00006D6533	105	54	1.0745	0.32541807	0.120	no	1.0745	0.334339	0.158	no	Bos taurus ST7 antisense RNA 1 conserved region 2
URS00006DD98B	78	51	0.8067	-0.8603869	0.000	no	0.8067	-0.83543	0.001	no	Ovis aries ZEB2 antisense RNA 1 conserved region 4
URS00006FC8F0	99	51	0.8301	-0.1825912	0.002	no	0.8301	-0.20831	0.015	no	Bos taurus MEST intronic transcript 1, antisense RNA conserved region
URS00006FECED	151	84	0.7746	-0.0439212	0.006	no	0.7746	-0.06789	0.029	no	Bos taurus Non-coding RNA BC040587
URS0000709DD8	124	0	0	0	0.000	no	0	0	0.004	no	Bos taurus GNAS antisense RNA 1 conserved region 5
URS000070AAE0	109	0	0	0	0.000	no	0	0	0.004	no	Bos taurus SOX2 overlapping transcript exon 4
URS00007294CE	76	57	0.5418	-0.2044881	0.001	no	0.5418	-0.15935	0.011	no	Ovis aries ZNRD1 antisense RNA 1 conserved region 2
URS000075E02A	947	84	0.5671	-0.2743852	0.001	no	0.5671	-0.26996	0.009	no	Bos taurus uncharacterized LOC100848574 (LOC100848574), transcript variant 3, long non-coding RNA.

Table S3 Sequence conservation of BLV AS1 and Tax sequences

	AS1	AS1 3rd	AS1 no overlap	AS1 no overlap 3rd	Tax	Tax 3rd
**AS1**	**0.01485 (264)**	0.0286	0.3265	0.0198	0.2995	0.1298
**AS1 3rd**		**0.034 (88)**	0.4859	0.4595	0.0011	0.4105
**AS1 no overlap**			**0.0236 (72)**	0.2278	0.0382	0.9165
**AS1 no overlap 3rd**				**0.0521 (24)**	0.0035	0.1246
**Tax**					**0.0106 (930)**	0.0031
**Tax 3rd**						**0.0247 (170)**

Sequence conservation of BLV AS1 and Tax sequences using sequence data from 12 bovine and 28 ovine tumors supplemented with six publicly available whole genome BLV sequences from NCBI
AS1: sequence segment corresponding to the putative protein coding sequence of AS1
AS1 3rd: 3rd codon positions of the putative protein coding sequence of AS1
AS1 no overlap: segment corresponding to the putative protein coding sequence of AS1 that does not overlap with R3 or G4
AS1 no overlap 3rd: 3rd codon positions of the putative protein coding sequence of AS1 that does not overlap with R3 or G4
Tax: full length Tax coding sequence, Tax 3rd: 3rd codon positions of the segment of Tax that does not overlap with Rex
Diagonal: nucleotide diversity (= average difference per nucleotide site across all pairs of isolates) for the corresponding sequence segment, and – between brackets – number of corresponding nucleotide positions. Off-diagonal: p-value of the differences between corresponding nucleotide diversities.

Table S4 Oligos used

BLV genome coordinates correspond to the provirus found in the YR2 cell line (NCBI Accession: KT122858)

End point PCR oligos

Name	Forward 5'-3'	Reverse 5'-3'	Region
AS1-S/L	TCCCTTCTGTTTCCACAACG	CAGAGGACCGGAGATAGAGC	BLV:7121-8564
AS1-L	CCCCTAAACCCGATTCTGAT	CAGAGGACCGGAGATAGAGC	BLV:6263-8564
AS2	CCCAGTCTCTTCTGGTGTCC	CAGAGGACCGGAGATAGAGC	BLV:2782-8564
ACTB	GACAATGGTTCTGGCATGTG	TCCTTGATGTCACGCACAAT	chr11:50421140-50422030 (oviAri3)

Real Time PCR oligos for use with SYBR green

Name	Forward 5'-3'	Reverse 5'-3'	Region
Tax	GCCTTCAAATGCCCAAAGAAC	CAACCAACAACACTTGCCATC	BLV:4818-7267
Env-Tax-Rex-R3	TCTTGCTCCCGAGACCTT	CTAGCCACCGACGATTGTTT	BLV:262-4712
AS1	TCCCTTCTGTTTCCACAACG	CTTCTGGTGCCGCTAACTC	BLV:7121-8404
AS2	TTTCGAACGGGCACTACAG	CTTCTGGTGCCGCTAACTC	BLV:2732-8404
TBP	CTTGACCTAAAGACCATTGCAC	CCATCTTCCCAGAGCTGAATATC	chr8:90670093-90671593 (oviAri3)
HPRT1	GACCAGTCAACAGGCGACAT	CTTGACCAAGGCAAGCAAAG	chrX:95382926-95389315 (oviAri3)
U6	CTCGCTTCGGCAGCACATATAC	GGAACGCTTCACGAATTTGCGTG	U6 Small nuclear RNA
Tyr-tRNA	CCTTCGATAGCTCAGTTGGT	GTCCTTCGAGCCGGAATC	Tyrosine transfer RNA

PrimeTime qPCR Assays (Integrated DNA Technologies)

Name	Forward 5'-3'	Probe	Reverse 5'-3'	Region
AS1 cDNA	TCCCTTCTGTTTCCACAACG	/56-FAM/AAGCTCTTC/Zen/GGGATCCATTACCTGC/3IABkFQ/	CTTCTGGTGCCGCTAACTC	BLV:7121-8404
AS2 cDNA	TTTCGAACGGGCACTACAG	/56-FAM/CCGCCTTCT/Zen/CCCAGTCTCTTCT/3IABkFQ/	CTTCTGGTGCCGCTAACTC	BLV:2732-8404
TAX cDNA	GCCTTCAAATGCCCAAAGAAC	/56-FAM/TGATGATCG/Zen/GTTGTGGGCGTCTTC/3IABkFQ/	CAACCAACAACACTTGCCATC	BLV:4818-7267
RPS9 cDNA	TGACGCTGGATGAGAAAGAC	/56-FAM/CTGTTCGAA/ZEN/GGTAATGCCCTG/3IABkFQ/	CTTCAGGCCCAGGATGTAATC	chr14:60,263,509-60,268,300 (oviAri3)
TBP cDNA	CTTGACCTAAAGACCATTGCAC	/56-FAM/TGCCCGAAA/ZEN/TGCTGAGTATAATCCCAA/3IABkFQ/	CCATCTTCCCAGAGCTGAATATC	chr8:90670093-90671593 (oviAri3)
BLV DNA	CTTACAACGCTTCCTCCATGAC	/56-FAM/ACGCTCACC/ZEN/TGGTCAGAATTGGTT/3IABkFQ/	GGCGGGAGAGCCATTTATTT	BLV:5806-5916
RPS9 DNA	TGGATGTTGGTATTGGGTGG	/56-FAM/TTCTGGTCT/ZEN/CAGGGAAACTTGCTGG/3IABkFQ/	CCCTGTGCCATCTCTTTGTC	chr18:63386124-63386267 (BosTau6)

Table S4 continued

Oligos for Luciferase assays

Name	Forward 5'-3'	Reverse 5'-3'	Region
5'LTR	TGACGCTAGCTGTATGAAAGATCATGCCGACCT	TGACAAGCTTACGAGCCCCCAATTGTTT	BLV:1-544
3'LTR	TGACAAGCTTTGAGGGGGAGTCATTTGTATG	TGACGCTAGCTGTTTGCCGGTCTCTCCT	BLV:8175-8720
3'LTRΔ	TGACAAGCTTGCTGACCTCACCTGCTGATA	TGACGCTAGCTGTTTGCCGGTCTCTCCT	BLV:8342-8720
mut DAS	CCTCTGACCGTCTCCATCAGGACTCTCTCTCTTG	CAAGAGAGAGAGTCCTGATGGAGACGGTCAGAGG	BLV:370-403/8559-8592
mut IRF	GTTTCCTGTCTTACAGTCTGTGTCTCGCGGCCCGC	GCGGGCCGCGAGACACAGACTGTAAGACAGGAAAC	BLV:450-484/8639-8673

Red bases = restriction recognition site

Oligos for producing altered pBLV344

Name	Forward 5'-3'	Reverse 5'-3'	Region	Additional info
up_miR	CCACCCTGGTACTCTTCCAA	TGACACGCGTGTGAAACATGGGGGTGTCA	BLV:3268-6388	Region upstream of miRs
down_miR	TGACACGCGTTAGACCCCCTAACGGAGGTT	AAGCAGAAAGAACGCTCGAA	BLV:6943--6962 & *chr12:59,152,995-59,153,014 (oviAri3)	Region downstream of miRs
amp_lig	CCACCCTGGTACTCTTCCAA	GCGGGAGAGCCATTTATTTT	BLV:3268-8168	Amplifies ligated DNA
amp_miR	TGACACGCGTGCACCCTCAGGCTGTGG	TGACACGCGTGAACCCGGGGCCTTG	BLV:6389-6942	Amplifies miR region

Red bases = restriction recognition site, *pBLV344 contains flanking DNA derived from the host

Oligos for 5'RACE

Description	Oligo 5'-3'
GeneRacer 5′ Nested With Nextera Forward	**TCGTCGGCAGCGTCAGATGTGTATAAGAGACAG**GGACACTGACATGGACTGAAGGAGTA
5'RACE AS1 + Nextera Reverse	**GTCTCGTGGGCTCGGAGATGTGTATAAGAGACAG**GGCTGCCTCTGCATCTTCTA
5'RACE AS2 + Nextera Reverse	**GTCTCGTGGGCTCGGAGATGTGTATAAGAGACAG**CCCAGTCTCTTCTGGTGTCC

Bold = region corresponding to either the nextera reverse or forward

Oligos for 3'RACE with GeneRacer oligo

Description	Oligo 5'-3'
GeneRace3	GCTGTCAACGATACGCTACGTAACGGCATGACAGTGTTTTTTTTTTTTTTTTTT
PCR1 GeneRacer3	GCTGTCAACGATACGCTACGTAACG
PCR1 BLV LTR (8489-8508)	GTGGTTTGCCTTACCTGACC
PCR2 GeneRacer3 nest with Nextera Forward	TCGTCGGCAGCGTCAGATGTGTATAAGAGACAGCGCTACGTAACGGCATGACAGTG
PCR2 BLV LTR (8405-8424)	AGGGTCTCAGGAGGAGAACG

Bold = region corresponding to either the nextera reverse or forward

Table S4 continued

Oligos for 3'RACE with oligo dT + Nextera

Description	Oligo 5'-3'
Oligo dT tailed with Nextera reverse	GTCTCGTGGGCTCGGAGATGTGTATAAGAGACAGTTTTTTTTTTTTTTTTTTV
PCR1 BLV LTR (8489-8508)	GTGGTTTGCCTTACCTGACC
PCR1 Nextera reverse	**GTCTCGTGGGCTCGGAGAT**
PCR2 BLV LTR (8405-8424)	AGGGTCTCAGGAGGAGAACG
PCR2 Nextera reverse	**GTCTCGTGGGCTCGGAGAT**

Bold = region corresponding to either the nextera reverse or forward

Nextera Based oligos annealed together and ligated to fragmented 3'RACE PCR

Description	Oligo 5'-3'
Nextera Forward	TCGTCGGCAGCGTC**AGATGTGTATAAGAGACAG**T
Nextera Reverse	/5Phos/**CTGTCTCTTATACACATCT**CCGAGCCCACGAGAC

Bold = region of complementarity in oligos

Oligos for 3'RACE with GeneRacer oligo primer adjacent to end of AS1-L

Description	Oligo 5'-3'
BLV (5393-5412) + Nextera Reverse	**GTCTCGTGGGCTCGGAGATGTGTATAAGAGACAG**CTGACAGAGGGAACCCAGTC
GeneRacer3 nest with Nextera Forward	TCGTCGGCAGCGTCAGATGTGTATAAGAGACAGCGCTACGTAACGGCATGACAGTG

Oligos for 3'RACE using GeneRacer oligo for MinION sequencing

Description	Oligo 5'-3'
GeneRace3	GCTGTCAACGATACGCTACGTAACGGCATGACAGTGTTTTTTTTTTTTTTTTTT
PCR1 GeneRacer3	GCTGTCAACGATACGCTACGTAACG
PCR1 BLV LTR (8489-8508)	GTGGTTTGCCTTACCTGACC
PCR2 GeneRacer3	CGCTACGTAACGGCATGACAGTG
PCR2 BLV LTR (8405-8424)	**GGTGCTGAAGAAAGTTGTCGGTGTCTTTGTGTTAACCT**AGGGTCTCAGGAGGAGAACG

Bold = MinION barcode 1

Table S4 continued

Oligos for modified 5’ RACE to detect RNA cleavage

Description PCR1 oligos	Oligo 5'-3'
GeneRacer 5′ Primer	CGACTGGAGCACGAGGACACTGA
miR1-RACE (BLV:6357-6376)	TCGACCCTGCCCTTGACACC
miR2-RACE (BLV:6454-6473)	CTTGCTGCCAGCGCCGAGTT
miR3-RACE (BLV:6584-6603)	GCCCTCCCGTTGCCTGTGAC
miR4-RACE (BLV:6682-6703)	GGGCGATTTCTTGCAGCTGTGC
miR5-RACE (BLV:6803-6827)	TACGCCCTGTTGCACACCCTTTCTA

Description PCR2 oligos	Oligo 5'-3'
GeneRacer 5′ Nested Primer with Nextera Forward	**TCGTCGGCAGCGTCAGATGTGTATAAGAGACAG**GGACACTGACATGGACTGAAGGAGTA
miR1-Nest + Nextera Reverse	**GTCTCGTGGGCTCGGAGATGTGTATAAGAGACAG**CCATGTTTCACGCACCCTCA
miR2-Nest + Nextera Reverse	**GTCTCGTGGGCTCGGAGATGTGTATAAGAGACAG**GCCCTACCCTGAGCCTCTCTG
miR3-Nest + Nextera Reverse	**GTCTCGTGGGCTCGGAGATGTGTATAAGAGACAG**CCTCTCTCACTTCTGCTTCACCA
miR4-Nest + Nextera Reverse	**GTCTCGTGGGCTCGGAGATGTGTATAAGAGACAG**GCAGCTGTGCTAAGCGAGAGG
miR5-Nest + Nextera Reverse	**GTCTCGTGGGCTCGGAGATGTGTATAAGAGACAG**ATCTCAGCTCGCACCCCAAG

Bold = region corresponding to either the nextera reverse or forward

Oligos for High-throughput sequencing of BLV integration sites

Description Oligos annealed to make adapter	Oligo 5'-3'
Linker-Long	TCATGATCAATGGGACGATCAGTCTCGTGGGCTCGGAGATGTGTATAAGAGACAGNNNNNNNNCGCTCTTCCGATCT
Linker-Short	/5Phos/GATCGGAAGAGCGAAAAAAAAAAAAA

Description PCR1 oligos	Oligo 5'-3'
Link-PCR1	TCATGATCAATGGGACGATCAGTC
BLV-5'END	GCAGCTGACGTCTCTGTCTG
BLV-3'END	AGCTCTATCTCCGGTCCTCT

Description PCR2 oligos	Oligo 5'-3'
Link-PCR2	GTCTCGTGGGCTCGGAGAT
BLV-5'END-2	TCGTCGGCAGCGTCAGATGTGTATAAGAGACAGNNNNNNNGGCTCCTAGGTCGGCATGAT
BLV-3'END-2	TCGTCGGCAGCGTCAGATGTGTATAAGAGACAGNNNNNNNCTAGCGGCCAGGAGAGACC

Table S4 continued

Locked nucleic acid antisense oligos for AS transcript knockdown

Name	Oligo 5'-3'	Region
BLV_AS_LNA1	CCAAGTGTGCACAATA	BLV:6994-7009
BLV_AS_LNA2	TACCTGCTGATAAAAC	BLV:7215-7217 & 8353-8365 covers AS1 spice site
BLV_AS_LNA3	AGCGTTCTCCTCCTGAG	BLV:8403-8419 Common to AS1 & AS2

Text S1 Extended materials and methods

Ovine/bovine samples and cell lines

The primary ovine tumors were part of an existing BLV induced tumor collection maintained at -80°C. They were derived from sheep experientially infected with BLV at the acute stage of the disease, where tumor development had occurred 15 to 48 months post infection. These tumors have been described previously [1, 2]. The bovine samples were part of a tumor collection sourced from Belgium, France, United States and Japan maintained at -80ºC and described previously [2]. Tumors samples were B-cell lymphoid masses that develop during natural BLV infection. Consequently, the interval between BLV infection and tumor development is not known.

The asymptomatic sheep samples were obtained from animals infected with the molecular clone pBLV344 following experimental procedures outlined in Willems et al [3] and were approved by the University of Saskatchewan Animal Care Committee, following Canadian Council on Animal Care Guidelines (Protocol #19940212). The animals were housed at the Vaccine and Infectious Disease Organization (VIDO-Intervac; Saskatoon, SK. Canada). Blood was collected in EDTA Vacutainers (BD, NJ, USA) and peripheral blood monocnuclear cells (PBMCs) were isolated by layering buffy coat cells on a one-step Percoll gradient [4].

The ovine B-cell tumor cell lines YR2 and L267 were derived from the M395 B-cell leukemia and the T267 B-cell lymphoma respectively. Both cell lines share the same proviral integration site as the primary tumor from which they were derived and do not express viral mRNAs originating in the 5'LTR and have been described previously [5, 6]. $L267_{LtaxSN}$ and $YR2_{LtaxSN}$ are the products of transduction of the L267 and YR2 cell lines with the pLTaxSn retroviral vector, producing constitutive expression of the viral transactivator Tax and as a consequence constitutive expression of the viral mRNAs [7]. The lymphoid cell suspensions were cultured in OPTIMEM medium (Invitrogen) supplemented with 10% FCS, 1 mM sodium pyruvate, 2 mM glutamine, MEM nonessential amino acids solution 1X (Gibco) and 100 μg/mL kanamycin at a concentration of 10^6 cells/mL. HeLa cells were procured from the American Type Culture Collection (ATCC), while the 293T cells were a gift from F. Kanshanchi (George Mason University, Manassas, VA). HeLa and 293T cells were cultured in DMEM supplemented with 10% FCS, L-glutamine and penicillin/streptomycin.

RNA sequencing

Total RNA was extracted using either TRIzol (Life Technologies) followed by treatment with turbo DNAse (Life Technologies) or with Qiagen AllPrep DNA/RNA/microRNA kit. Using 1ug of RNA, strand specific, ribosomal RNA depleted libraries were prepared with the Illumina TruSeq Total RNA stranded kit. The resultant libraries were assesed on an Agilent Bioanalyzer DNA 1000 and by qPCR with the KAPA kit (KAPA biosystems) followed by sequencing on an Illumina HiSeq 2000 (2 x 100bp paired-end reads), generating approximately 60 million raw paired-end reads per library. Small RNA libraries were prepared using the Illumina TruSeq Small RNA Library Preparation Kit and sequenced on an Illumina NextSeq 500 (75bp single reads). Custom host-provirus reference genome builds were generated using the bovine UMD3.1 [8] or ovine OAR3.1 [9] and the proviral genome sequence for BLV YR2 (NCBI Accession: KT122858) and the resultant RNA reads were mapped to the appropriate genome using STAR

(v2.3.1.u) [10] and BWA [11]. Sorting, indexing and separating of the sense from the antisense was carried out with SAMtools [12] and BAMtools [13]. Read quantification and read count normalization was carried out with FeatureCounts [14] and R packages DESeq2 [15] respectively. Transcript quantification was also assessed with RSEM [16]. The percentage of AS1-S vs AS1-L transcripts observed was calculated by counting the reads aligning to the AS1-L region (5237-6895) and normalized to the region length (1658bp). Reads aligning to the region in common to AS1-S & AS1-L (6895-7217) were normalised to the length (322) followed by subtraction of the reads from the AS1-L. The ratio of AS1-S compared to AS1-L was then computed via: AS1.S.norm / (AS1.S.norm+AS1.L.norm). This was computed based on all the ovine and bovine samples as well as the YR2 and L267 cell lines. The Integrative Genomic Viewer (IGV) was used for visulisation of aligned sequences [17]. Reads orignating from either of the two BLV LTRs aligned to both the 5' and 3' LTRs due to the identical sequences of both LTRs.

End point PCR

RNA and DNA was extracted using the Qiagen AllPrep DNA/RNA/microRNA kit. Reverse transcription was carried out using SuperScript III Reverse Transcriptase (Life technologies), with subsequent RNase H treatment (New England Biolabs) and primed with random hexamers. Each PCR reaction contained 1ul cDNA, 3pmol forward and reverse primer, 0.1ul dNTPs (10 mM), 0.075ul GoTaq Hot Start Polymerase (Promega), 2ul Q5 buffer and 6.2ul H2O. Annealing and extension conditions were based on the manufacturer's recommendations, with a total of 35 cycles used. PCR products were visualized on a 1.5% agarose gel. PCR primers used are listed in Table S4.

High-throughput sequencing of BLV integration sites

To ensure that asymptomatic animals were in a polyclonal stage of infection we carried out high throughput sequencing of proviral integration sites. The method used is similar to that outlined by [18, 19], while incorporating a number of changes to increase sensitivity and facilitate sample multiplexing and is detailed in (Rosewick et al, submitted). Briefly, 5ug of DNA is sheared in a Bioruptor Pico (Diagenode) following the manufacturer's instructions for fragments of ~1000bp. Samples are end repaired and dA tailed with the NEBNext Ultra End Repair/dA-Tailing Module (New England Biolabs) followed by ligation to a linker containing the Nextera Reverse sequence and a upstream sequence to facilitate nested PCR. Nested PCR was carried out with primer matching the linker and the BLV LTRs. Nextera XT indexes (Illumina) were added followed by sequencing on a Illumina MiSeq instrument with 2x75bp reads (Reagent Kit v3) or 2x150bp (Reagent Kit v2). The resultant paired-end reads were aligned to the host-provirus hybrid genome with BWA. Reads were trimmed based on average base quality (>= 30) and paired end reads spanning the LTR-host junction (Read 1: 8 nucleotides mapping to the LTR; Read 2: host alignment with maximum 3 mismatches) were extracted based on soft-clipped sequences and mis-paired reads. Read numbers were determined for each proviral integration site using in-house R and Perl scripts. Clone abundance was calculated as an average for the 3' and 5'LTRs.

Proviral load quantification

DNA was extracted with the AllPrep DNA/RNA/microRNA (Qiagen). Proviral load was quantified using PrimeTime qPCR Assays (Integrated DNA Technologies) targeting the BLV

provirus and RPS9 in the host genome for normalization (Listed in Table S4). Reactions were carried out in a 10ul volume with 50ng of template DNA, 1 X Taq Man Universal PCR Master Mix, No AmpErase UNG (Thermo Scientific) and a 1 X dilution of the appropriate PrimeTime assay mix. Thermocycling conditions were 10 min at 95°C, with 40 cycles at 95°C for 15 sec and 60°C for 1 min. Standard curves were generated using serial dilutions of DNA from the YR2 cell line and proviral load in % PBMCs calculated as (Sample Average Quantity) x 2 / (Sample RPS9) * 100. (In the YR2 cell line the chromosome into which the BLV provirus integrated has been duplicated, as a consequence each cell carries two provirus copies.)

Identification of 5' and 3' ends with RACE

Total RNA was extracted from the YR2 cell line with TRIzol (Life Technologies) and treated with turbo DNAse (Life Technologies). The GeneRacer Kit (Life Technologies) was used to amplify 5' ends of both BLV antisense transcripts with a modified protocol. The manufacturer's protocol was followed until the point of the 1st PCR. At this stage primers tailed with a sequence corresponding to the Nextera forward and reverse were used (adapting the protocol described for the perpetration of 16S Ribosomal RNA Gene Amplicons). The addition of the Nextera sequences to the ends of the PCR products facilitated the addition of Illumina adapters and indexes using Nextera XT primers (Illumina), subsequent pooling and high throughput sequencing. In the modified protocol the initial round of PCR was carried out using 2.5 ul of the appropriate cDNA, 1.25ul (10µM) primer tailed with the Nextera forward or reverse (listed in Table S4), 0.5ul dNTPs (10 mM), 0.25ul Q5 High-Fidelity DNA Polymerase (New England Biolabs), 5ul Q5 buffer and 14.5ul H2O. A total of 35 cycles were carried out, extension and annealing times were based on the manufacturer's recommendations. The resultant PCR product was cleaned up using the QIAquick PCR purification kit (Qiagen), Illumina sequencing adapters and indexes were added via PCR using Illumina Nextera XT primers. The reaction was cleaned up with the QIAquick PCR purification kit (Qiagen) and the resultant library concentration was determined with PicoGreen (Invitrogen). Libraries were pooled with additional Nextera based libraries followed by sequenced 2x75bp on a Illumina MiSeq instrument.

For 3' RACE three slightly different approaches were taken. In all cases cDNA was produced from ~1750ng of total RNA using SuperScript III Reverse Transcriptase (Life technologies), with subsequent RNase H treatment (New England Biolabs). The primer used for cDNA priming was either the GeneRacer oligo dT or an oligo dT tailed with the Nextera reverse sequence attached (see Table S4 for oligos). The first approach to 3'RACE is similar to that that outlined in Rosewick et al, submitted. Briefly the 1st PCR used 5ul of cDNA as template, primers matching the BLV AS exon 1 and the Nextera reverse and Q5 High-Fidelity DNA Polymerase (New England Biolabs). Annealing temperature was 66°C, with a 4 min extension and a total of 25 cycles. Following cleaning with 1.8X AMPure XP beads (Beckman Coulter), a second semi-nested PCR was then carried out using the same primer matching the Nextera reverse and a second primer in the BLV AS exon 1, with the same cycling conditions as above. The resultant PCR product was then sheared in a Bioruptor Pico (Diagenode) following the manufacturer's instructions for fragments of ~400bp. End repair and dA tailing with the NEBNext Ultra End Repair/dA-Tailing Module (New England Biolabs) was followed by cleaning with AMPure XP beads (Beckman Coulter) and ligation to 60pmol annealed oligos corresponding to the Nextera forward and reverse sequences with T4 DNA ligase (Ligase New England Biolabs). The DNA was again cleaned with 0.8X AMPure XP beads and Nextera XT indexes (Illumina) were added

by PCR, the indexed libraries were again cleaned with 0.8X AMPure XP beads and quantified by PicoGreen (Invitrogen). The resultant libraries were mixed with additional Nextera based libraries and sequined at either 2x75bp (Reagent Kit v3) or 2x150bp (Reagent Kit v2) on a Illumina MiSeq instrument. Reads from both approaches were mapped and visualized in the same manner outlined for the RNA sequencing data.

The second approach to 3'RACE utilized cDNA primed with the GeneRacer oligo dT. In this approach only a single round of PCR was carried out with a primer matching the GeneRacer oligo tailed with the Nextera Forward sequence and a second primer just upstream of the potential poly A site in AS1-L tailed with the Nextera Reverse sequence (see Table S4 for oligos). The PCR used 2.5ul of cDNA, Q5 High-Fidelity DNA Polymerase (New England Biolabs), with an annealing temperature of 67°C and a 45 sec extension and a total of 35 cycles. The reaction was cleaned up with a MiniElute column (Qiagen). Addition of the Nextera XT indexes (Illumina) quantification and sequencing on the Illumina MiSeq instrument was carried out in the same manner as described above.

The final approach to the 3'RACE utilized the MinION's (Oxford Nanopore Technologies) ability to produce long reads to observe nearly full length AS1-L transcripts. Starting with 2.5 ul of cDNA (primed with the GeneRacer oligo dT), a 1st PCR was carried out using LongAmp Taq DNA Polymerase (New England Biolabs). Cycling conditions followed the manufacture recommendations with an annealing temperature of 60°C, an extension time of 11 min and 35 cycles. The resultant PCR product was cleaned up twice using 0.8X AMPure XP beads to ensure the removal of small fragments. A nested PCR was then carried out, again following the manufacturer's recommendations for a 50ul reaction with 1ul of the previous PCR as template. Annealing temperature was 60°C, with an extension time of 3 min and 15 cycles. The resultant PCR was divided, with 10ul loaded on a 1% agarose gel stained with SYBR Safe (Thermo Fisher) and the remaining 40 ul loaded on a second 1% agarose, both gels were also loaded with 10kb to 200bp DNA ladder. The gels were run in parallel in the same electrophoresis tank, the stained gel was visualized under UV light and used to determine when the fragments had separated sufficiently. Using this gel as a guide the portion of the unstained gel containing fragments between ~1.5kb and 5kb was excised and purified using the MinElute Gel Extraction Kit (Qiagen). This was done to avoid exposing the DNA fragments to UV light as they will be directly sequenced on the MinION device. Using 1ug of the purified size selected DNA as template a library was prepared following the Amplicon sequencing v11 protocol, with the SQK-MAP-006 Nanopore sequencing kit followed by analysis using the SQK-MAP-006 protocol. FASTA sequences for both the 2D reads (where both strands of the fragment are sequenced) and 1D reads (only one strand sequenced) were extracted with Poretools [20]. The sequences were mapped to the same custom hybrid Ovine/BLV genome described above in the RNA sequencing section using the option for Nanopore reads in BWA [11], followed by processing with SAMtools [12] and visualization in IGV [17].

Luciferase assays

The BLV LTR utilized was derived from the pBLV344 plasmid described previously [3]. The various constructs were cloned into a pGL3 basic luciferase reporter plasmid. Primers used to amplify the LTR from pBLV344 and for site directed mutagens are listed in Table S4. HeLa cells were transfected in triplicate using lipofectamine 2000 (Life technologies) with 400ng of the appropriate construct in addition to 10ng Renilla control plasmid. In cases where the size of

insert differed, the amount of DNA transfected was adjusted to ensure equal copy numbers for each construct. Tax expression constructs used were previously described [6]. Forty-eight hours post transfection the cells were processed using the Dual-Glo Luciferase Assay System (Promega) following the manufacturer's instructions. Statistical significance was assessed by Tukey's multiple comparisons test, carried out using the Prism software (Graphpad), with a p-value < 0.05 considered statistically significant.

Protein coding potential and nucleotide diversity

Coding potential was assessed using the Coding-Potential Assessment Tool (CPAT) [21]. The limited number of annotated bovine lncRNA made the construction of a robust bovine specific model impractical. Instead full length sequence of the BLV antisense transcripts, five protein coding genes from HLTV-1 (including HBZ) and BLV along with 220 bovine and ovine lncRNAs obtained from rnacentral.org [22] were collected. These sequences were then analyzed with the CPAT web server [23] using precompiled models based on human and mouse training sets [21].

To determine the sequence conservation of the potential open reading frame in AS1-S/L we examined the region in a number of BLV genomes. BLV consensus sequences were extracted from ovine and bovine aligned RNA-Seq data (STAR) using a samtools mpileup, bcftools and seqtk based custom script. An additional six BLV whole genome sequences were downloaded from NCBI (http://www.ncbi.nlm.nih.gov/nuccore) and included in the analysis. Calculation of percentage of identity and significance was completed in the same way as described in Rosewick et al [1].

Sub-cellular location of BLV antisense transcripts

To produce cytoplasmic and nuclear fractions for YR2 we followed the method outlined in Weil et al [24]. Briefly, ~$40x10^6$ cells were washed twice in PBS, 200ul of fractionation buffer (Tris HCl 10mM, NaCl 140mM, MgCl2 1.5mM, EDTA 10mM, NP40 0.5%, RNaseOUT 100U/ml was then added and the cells were incubated on ice for 5 minutes. The resultant lysate was centrifuged for 5 min at 4^oC and the supernatant transferred to a new tube (retaining the pellet), followed by centrifugation at full speed for one minute. The supernatant was again transferred to a new tube and 1 ml Trizol added (cytoplasmic fraction). For the pellet, 200ul of fractionation buffer was added and the pellet mixed by pipetting, followed by centrifugation for 5 min at 4^oC. Supernatant was discarded and the pellet resuspended in 200ul of fractionation buffer followed by disruption in a TissueLyser II (Qiagen), with the subsequent addition of 1 ml of Trizol (nuclear fraction). RNA was extracted from both fractions and the resultant RNA was then treated with turbo DNAse (Life Technologies). RNA from both fractions was sequenced and the reads mapped in the same manner as outlined above. Enrichment in nuclear and cytoplasmic fractions was computed as follows : Paired-end RNA-Seq reads were aligned on the ovine (OAR3.1) with STAR [10]. Read counts for the different RNA species (snRNA, snoRNA, tRNA,..) were computed using featureCounts and ENSEMBL v74 annotation. To account for sequencing depth variation across samples, read count normalization was performed using DESeq2 [15]. Finally, abundance of each RNA species was computed by comparing the total normalized read count of each RNA species.

Real time PCR based absolute quantification (see Table S4 for oligos) was completed on the ABI Prism 7900HT Sequence Detector System (Applied Biosystems) using ABsolute Blue QPCR SYBR Green ROX Mix (Thermo Scientific). PCR products were cloned into a TOPO TA plasmid (Life Technologies) and then diluted to the appropriate concentration to produce a standard curve. Relative enrichment of the transcript was calculated in the same manner as Kobayashi-Ishihara et al [25] % enrichment = RNA level in nucleus / combined RNA levels for nuclear and cytoplasmic fraction × 100.

Identifying products of RISC mediated cleavage

RNA was extracted from the YR2 cell line and PBMCs from an asymptomatic sheep (17 months post-inoculation with pBLV344) using Trizol (Life Technologies) and treated with turbo DNAse (Life Technologies). In the case of the cytoplasmic and nuclear RNA YR2 cells were fractionated following the method outlined above. The procedure for identifying the products of slicing was based on Davis et al [23]. The RNA Oligo from the gene RACE kit (Promega) was ligated to 3.8ug of total RNA using 1ul 10x Ligase Buffer, 1ul 10mM ATP, 1ul RNaseOut, 1ul T4 RNA ligase in a total volume of 10ul. The mix was incubated for 1hr at 37°C followed by precipitation and resuspension of the RNA in 10ul of H2O. Revere transcription was carried out with Revert Aid Premium Reverse Transcriptase (Thermo Scientific) and random hexamers. PCR was initially carried out in five separate reactions with the common forward Gene Racer 5' primer in combination with a primer designed to identify the slicing of one of the BLV microRNAs. The PCR reaction contained 2ul cDNA, 0.3ul (10 µM) forward and reverse primer, 0.1ul dNTPs (10 mM), 0.1ul Q5 High-Fidelity DNA Polymerase (NEB), 2ul Q5 buffer and 5.2ul H2O (see Table S4 for oligos). A total of 30 cycles were performed using annealing and extension conditions recommended by the manufacturer. A nested PCR was then carried out using tailed primers carrying the Nextera forward and reverse sequences. The resultant PCR products were pooled and cleaned up with the QIAquick PCR purification kit (Qiagen). As outlined above the Illumina adapters and indexes were added using Nextera XT indexes (Illumina). The resultant libraries were paired-end sequenced 2 x 75bp on an Illumina MiSeq using the Reagent Kit v3. To determine the precise position of the free 5' end of the RNA we took the 30bp sequence of the RNA oligo and concatenated it to a sliding window of 31bp sequences from the BLV microRNA region. Starting at position 6357 a total of 554 61bp hybrid sequences were created. A BASH script was then used to search the FASTA files of each library for perfect matches with the concatenated sequences, thereby identifying the position and frequency of RNA cleavage. The high throughput sequencing reads were also mapped to the ovine-BLV hybrid genome using BWA and visualized in IGV.

Deleting and inverting the BLV microRNAs

The deletion and inversion of BLV microRNAs was carried out on the wild type molecular clone pBLV344 [3]. PCR primers were designed to amplify a 3kb region just upstream of the BLV microRNA cluster and a 1.7 kb region downstream of the microRNAs that included the entire terminal portion of the BLV provirus. The recognition sequence for the MluI restriction enzyme was used to tail the reverse primer of the upstream primer pair and the forward primer of the downstream pair (see Table S4). PCR was completed using Phusion High-Fidelity DNA Polymerase (NEB) with the pBLV344 plasmid as template. PCR products were digested with MluI (NEB) and then ligated together using Promega T4 DNA ligase. The ligation product was

run on a 1% gel and the band of the appropriate size (4.7kb) cut out and purified. The altered BLV fragment was then introduced into the pBLV344 plasmid using the BspEI (NEB) and NheI (Promega) restriction enzymes. This resulted in a provirus lacking the BLV microRNAs with a MluI recognition site incorporated in their place. The inverted plasmid was created by amplifying the BLV microRNA cluster using primers tailed with the MluI recognition site. The resultant PCR product and the pBLV344 plasmid lacking the microRNAs (with MluI site added) were digested with MluI and the PCR product inserted into the provirus by ligation. Sanger sequencing was used to check the orientation of the reinserted region and indentify a plasmid where the BLV microRNA orientation was inverted. The resultant constructs were transfected into HeLa cells followed by RNA extraction, using the same protocol outlined above.

Antisense knock down with locked nucleic acid antisense oligos (LNAs)

The BLV cell lines YR2 and L267 are refractory to transfection, consequently it is not possible to knock down the expression of the BLV antisense transcripts using more conventional approaches such as siRNAs. We therefore employed locked nucleic acid antisense oligos (Exiqon), introduced via unassisted uptake [26], following the manufacturer's recommendations. A half million YR2 and L267 cells/well were plated in 24 well plates in a final volume of 500 ul. Each cell line was treated in duplicate with a mix of three LNAs that targeted different parts of AS1 (Table S4). Two different final concentrations were tested, 5 μM and 10 μM. As a negative control both cell lines were treated in duplicate with the LNA longRNA GapmeR Negative control A (Exiqon), at a final concentration of 10 μM and a mock treatment using 25 ul of H20 was also carried out. Cells were incubated for 5 days and cell numbers were then estimated with a Bio-Rad TC20 Automated Cell Counter. DNA and RNA were extracted using the Qiagen AllPrep DNA/RNA/microRNA kit. cDNA was produced using SuperScript III (Invitrogen) and random hexamers. Real time PCR was carried out using PrimeTime qPCR Assays (Integrated DNA Technologies) and Taq Man Universal PCR Master Mix, No AmpErase UNG (Thermo Scientific) on a ABI 7900HT Fast Real-Time PCR System. Data analysis was carried out with the qbase+ software (Biogazelle), with graphs and statistical analysis carried out using the Prism software (Graphpad). Expression levels for each sample were normalized against RSP9 and TBP levels. To combine the expression levels for both cell lines, expression levels were first scaled to the LNA Negative control of each cell line. P values of ≤ 0.05 were considered to be statistically significant after controlling for multiple testing. Statistical significance was assessed via a Kruskal-Wallis test and Dunn's multiple comparisons test, P values of ≤ 0.05 were considered to be statistically significant.

References for extended materials and methods

1. Rosewick N, Momont M, Durkin K, Takeda H, Caiment F, Cleuter Y, Vernin C, Mortreux F, Wattel É, Burny A, Georges M, Van den Broeke Anne: **Deep sequencing reveals abundant noncanonical retroviral microRNAs in B-cell leukemia/lymphoma.** *Proceedings of the National Academy of Sciences* 2013, **110**:2306–2311.

2. Kettmann R, Deschamps J, Cleuter Y, Couez D, Burny A, Marbaix G: **Leukemogenesis by bovine leukemia virus: proviral DNA integration and lack of RNA expression of viral long terminal repeat and 3' proximate cellular sequences.** *Proceedings of the National Academy of Sciences* 1982, **79**:2465–2469.

3. Willems L, Kettmann R, Dequiedt F, Portetelle D, Vonèche V, Cornil I, Kerkhofs P, Burny A, Mammerickx M: **In vivo infection of sheep by bovine leukemia virus mutants.** *Journal of Virology* 1993, **67**:4078–4085.

4. Mena A, Nichani AK, Popowych Y, Godson DL, Dent D, Townsend HGG, Mutwiri GK, Hecker R, Babiuk LA, Griebel P: **Innate immune responses induced by CpG oligodeoxyribonucleotide stimulation of ovine blood mononuclear cells.** *Immunology* 2003, **110**:250–257.

5. Kettmann R, Cleuter Y, Gregoire D, Burny A: **Role of the 3' long open reading frame region of bovine leukemia virus in the maintenance of cell transformation.** *Journal of Virology* 1985, **54**:899–901.

6. Merimi M, Klener P, Szynal M, Cleuter Y, Bagnis C, Kerkhofs P, Burny A, Martiat P, Van den Broeke Anne: **Complete suppression of viral gene expression is associated with the onset and progression of lymphoid malignancy: observations in Bovine Leukemia Virus-infected sheep**. *Retrovirology* 2007, **4**:51.

7. Merimi M, Klener P, Szynal M, Cleuter Y, Kerkhofs P, Burny A, Martiat P, Van den Broeke Anne: **Suppression of viral gene expression in bovine leukemia virus-associated B-cell malignancy: interplay of epigenetic modifications leading to chromatin with a repressive histone code.** *Journal of Virology* 2007, **81**:5929–5939.

8. Zimin A, Delcher A, Florea L, Kelley D, Schatz M, Puiu D, Hanrahan F, Pertea G, Van Tassell C, Sonstegard T, Marcais G, Roberts M, Subramanian P, Yorke J, Salzberg S: **A whole-genome assembly of the domestic cow, Bos taurus**. *Genome Biol* 2009, **10**:R42–1641.

9. The International Sheep Genomics Consortium, Archibald AL, Cockett NE, Dalrymple BP, Faraut T, Kijas JW, Maddox JF, McEwan JC, Hutton Oddy V, Raadsma HW, Wade C, Wang J, Wang W, Xun X: **The sheep genome reference sequence: a work in progress**. *Animal Genetics* 2010, **41**:449–453.

10. Dobin A, Davis CA, Schlesinger F, Drenkow J, Zaleski C, Jha S, Batut P, Chaisson M, Gingeras TR: **STAR: ultrafast universal RNA-seq aligner**. *Bioinformatics* 2012, **29**:15–21.

11. Li H: **Aligning sequence reads, clone sequences and assembly contigs with BWA-MEM**. *arXiv preprint arXiv:13033997* 2013.

12. Li H, Handsaker B, Wysoker A, Fennell T, Ruan J, Homer N, Marth G, Abecasis G, Durbin R, 1000 Genome Project Data Processing Subgroup: **The Sequence Alignment/Map format and SAMtools**. *Bioinformatics* 2009, **25**:2078–2079.

13. Barnett DW, Garrison EK, Quinlan AR, Stromberg MP, Marth GT: **BamTools: a C++ API and toolkit for analyzing and managing BAM files**. *Bioinformatics* 2011, **27**:1691–1692.

14. Liao Y, Smyth GK, Shi W: **featureCounts: an efficient general purpose program for assigning sequence reads to genomic features**. *Bioinformatics* 2014, **30**:923–930.

15. Love MI, Huber W, Anders S: **Moderated estimation of fold change and dispersion for RNA-seq data with DESeq2**. *Genome Biol* 2014, **15**:550–21.

16. **RSEM: accurate transcript quantification from RNA-Seq data with or without a reference**. 2011:1–16.

17. Thorvaldsdóttir H, Robinson JT, Mesirov JP: **Integrative Genomics Viewer (IGV): high-performance genomics data visualization and exploration.** *Briefings in Bioinformatics* 2013, **14**:178–192.

18. Gillet NA, Gutiérrez G, Rodriguez SM, de Brogniez A, Renotte N, Alvarez I, Trono K, Willems L: **Massive Depletion of Bovine Leukemia Virus Proviral Clones Located in Genomic Transcriptionally Active Sites during Primary Infection**. *PLoS Pathog* 2013, **9**:e1003687.

19. Gillet NA, Malani N, Melamed A, Gormley N, Carter R, Bentley D, Berry C, Bushman FD, Taylor GP, Bangham CRM: **The host genomic environment of the provirus determines the abundance of HTLV-1-infected T-cell clones**. *Blood* 2011, **117**:3113–3122.

20. Loman NJ, Quinlan AR: **Poretools: a toolkit for analyzing nanopore sequence data**. *Bioinformatics* 2014, **30**:3399–3401.

21. Wang L, Park HJ, Dasari S, Wang S, Kocher JP, Li W: **CPAT: Coding-Potential Assessment Tool using an alignment-free logistic regression model**. *Nucleic Acids Research* 2013, **41**:e74–e74.

22. The RNAcentral Consortium: **RNAcentral: an international database of ncRNA sequences**. *Nucleic Acids Research* 2015, **43**:D123–D129.

23. Davis E, Caiment F, Tordoir X, Cavaillé J, Ferguson-Smith A, Cockett N, Georges M, Charlier C: **RNAi-Mediated Allelic trans-Interaction at the Imprinted Rtl1/Peg11 Locus**. *Current Biology* 2005, **15**:743–749.

24. Weil D, Boutain S, Audibert A, Dautry F: **Mature mRNAs accumulated in the nucleus are neither the molecules in transit to the cytoplasm nor constitute a stockpile for gene expression.** *RNA* 2000, **6**:962–975.

25. Kobayashi-Ishihara M, Yamagishi M, Hara T, Matsuda Y, Takahashi R, Miyake A, Nakano K, Yamochi T, Ishida T, Watanabe T: **HIV-1-encoded antisense RNA suppresses viral replication for a prolonged period.** *Retrovirology* 2012, **9**:38.

26. Stein CA, Hansen JB, Lai J, Wu S, Voskresenskiy A, Høg A, Worm J, Hedtjärn M, Souleimanian N, Miller P, Soifer HS, Castanotto D, Benimetskaya L, Ørum H, Koch T: **Efficient gene silencing by delivery of locked nucleic acid antisense oligonucleotides, unassisted by transfection reagents.** *Nucleic Acids Research* 2010, **38**:e3–e3.
